# Highly Selective Separation Intermediate‐Size Anionic Pollutants from Smaller and Larger Analogs via Thermodynamically and Kinetically Cooperative‐Controlled Crystallization

**DOI:** 10.1002/advs.202003243

**Published:** 2021-02-01

**Authors:** Yongsheng Mi, Chaofeng Zhao, Shaomin Xue, Ning Ding, Yao Du, Hui Su, Shenghua Li, Siping Pang

**Affiliations:** ^1^ School of Materials Science & Engineering Beijing Institute of Technology Beijing 100081 P. R. China

**Keywords:** crystallization, metal‐organic frameworks, organic analogs, organic anionic pollutants, separation

## Abstract

Selective separation of organic species, particularly that of intermediate‐size ones from their analogs, remains challenging because of their similar structures and properties. Here, a novel strategy is presented, cooperatively (thermodynamically and kinetically) controlled crystallization for the highly selective separation of intermediate‐size anionic pollutants from their analogs in water through one‐pot construction of cationic metal‐organic frameworks (CMOFs) with higher stabilities and faster crystallization, which are based on the target anions as charge‐balancing anions. 4,4′‐azo‐triazole and Cu^2+^ are chosen as suitable ligand and metal ion for CMOF construction because they can form stronger intermolecular interaction with *p*‐toluenesulfonate anion (Tsˉ) compared to its analogs. For this combination, a condition is established, under which the crystallization rate of a Tsˉ‐based CMOF is remarkably high while those of analog‐based CMOFs are almost zero. As a result, the faster crystallization and higher stability cooperatively endow the cationic framework with a close‐to‐100% selectivity for Tsˉ over its analogs in two‐component mixtures, and this preference is retained in a practical mixture containing more than seven competing (analogs and inorganic) anions. The nature of the free Tsˉ anion in the cationic framework also allows the resultant CMOF to be recyclable via anion exchange.

## Graphical abstract

A novel strategy, cooperatively (thermodynamically and kinetically) controlled crystallization for the highly selective separation of intermediate‐size anionic pollutants, is proposed. Using this strategy, *p*‐tosylate (Ts^−^) anion is effectively separated from its analogs of benzenesulfonate (Bs^−^) and *p*‐ethylbenzenesulfonate (Es^−^), and the selectivity is as high as 100%. The nature of the free anion also allows the resultant materials to be recyclable.

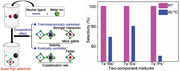

## 1 Introduction

Separation of organic species from their analogs is a vital step of industrial and wastewater treatment processes.^[^ ^1^ ^]^ However, due to their very similar structures and properties, separation of organic species from their analogs remains exceedingly difficult. One of the most‐used ways to separate such mixtures is by using porous materials with different pore sizes and shapes. Over the past decade, a large number of porous materials including porous organic polymers,^[^ ^2^ ^]^ hydrogen‐bonded organic frameworks,^[^ ^3^ ^]^ covalent‐organic frameworks (COFs),^[^ ^4^ ^]^ metal‐organic frameworks (MOFs)^[^ ^5^ ^]^ and organic coordination cages^[^ ^6^ ^]^ have been developed and investigated for the separation of gas or vaporized neutral species from their competing analogs such as C_2_H_2_/C_2_H_4_,^[^ ^7^ ^]^ C_2_H_4_/C_2_H_6_,^[^ ^8^ ^]^ C_3_H_6_/C_3_H_8_,^[^ ^9^ ^]^ C_3_H_4_/C_3_H_6_,^[^ ^10^ ^]^ C_4_H_6_/C_4_H_8_,^[^ ^11^ ^]^ and styrene/ethylbenzene,^[^ ^12^ ^]^ which exhibit significantly enhancing selectivities for target species via molecular sieving effect. Some of them have also been explored for the separation of anionic organic pollutants such as surfactants, biomolecules, and dyes from small inorganic anions in wastewater.^[^ ^13^ ^]^ In contrast, selective separation of these anions from their analogs in wastewater using porous materials has been scarcely reported possibly because i) the separation performances of such materials may greatly decrease in the presence of competing analogs;^[^ ^14^ ^]^ and ii) some porous materials such as MOFs and COFs suffer from poor stabilities in water.^[^ ^15^ ^]^ In particular, as the size exclusion mechanism of these porous materials cannot be applied to target species smaller than the pore apertures,^[^ ^16^ ^]^ separation of intermediate‐size organic anions from their smaller and larger analogs remains an important challenge. However, this separation is desirable to avoid environmental pollution and recovery and reuse these anions in highly pure form.

As an emerging subclass of crystalline MOFs, cationic MOFs (CMOFs) can be constructed through the coordination‐based self‐assembly of neutral nitrogen‐containing ligands and metal ions or clusters.^[^ ^17^ ^]^ In this case, the charge‐balancing anions are encapsulated into the pores of the cationic framework and are often free and uncoordinated to the metal centers. Based on the structural features of CMOFs, Custelcean's group has developed a one‐pot thermodynamically controlled crystallization for the separation of inorganic anionic pollutants from wastewater through the construction of more stable CMOFs based on the target anions as charge‐balancing anions via choosing suitable ligands and metal ions.^[^ ^18^ ^]^ Recently, our group has also used this strategy to selectively separate an organic pollutant anion (2,4,6‐trinitrophenolate) from wastewater.^[^ ^19^ ^]^ Advantageously, the above strategy employs water as a solvent, allowing the separation of pollutant anions from wastewater. Meanwhile, according to the shape and sizes of the target anions, the matched pores of the cationic frameworks can be constructed through self‐assembly between the ligands and metal ions, which is difficult to achieve for porous materials with pre‐existing rigid pores. More importantly, this technique usually affords single crystalline CMOFs, thus facilitating the observation of molecular interactions between the host frameworks and target guest anions by X‐ray crystallography and allowing to disclose the mechanism of selective separation. Despite these advantages, this strategy still suffers from poor selectivity for organic anions over their competing analogs with similar structures. This is not strong enough to distinguish a minor difference between these anions and their analogs.

On the other hand, one‐pot kinetically controlled crystallization has been widely employed to control the crystallization rates of MOFs, modulate their structure, shape, and size and improve their functions through a simple change of reaction parameters such as ligand, metal ions, temperature, solvent, and pH.^[^ ^20^ ^]^ For example, Zhou's group originally designed and synthesized several hybrid core‐shell MOFs (e.g., PCN‐222@Zr‐BPDC) with enhanced functionalities via one‐pot reaction by controlling the crystallization rate of original MOFs (e.g., PCN‐222 and Zr‐BPDC).^[^ ^20a^ ^]^ Kitagawa's group employed this strategy to create phase‐separated MOFs with unique gas adsorption properties.^[^ ^20b^ ^]^

**Figure 1:**
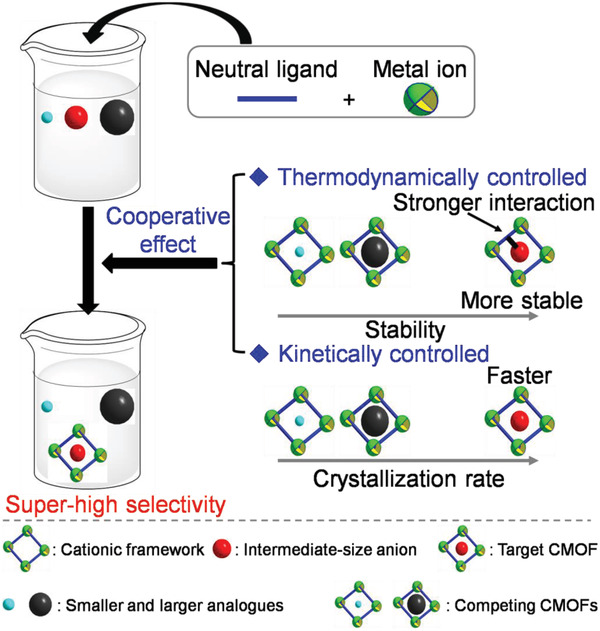
Separation of intermediate‐size anions from their smaller and larger analogs via cooperatively (thermodynamically and kinetically) crystallization strategy (this work).

Inspired by these works, we herein combined thermodynamically and kinetically crystallizations to develop a novel strategy for selective separation of more challenging pollutant anions, particularly intermediate‐size ones from their analogs in water through one‐pot construction CMOFs featuring higher stabilities and faster crystallization, and containing the target anions as charge‐balancing species. In this strategy (**Figure** **1**), according to the structures of the target anions, water‐soluble neutral ligands and metal ions would be strategically designed to make the intermolecular interaction between the resultant cationic frameworks and the target anions stronger than those between these frameworks and their analogs. In turn, this contributes to the construction of more thermodynamically stable CMOFs in water and thus improving the selectivities of the cationic frameworks for the target anions. On the basis of the designed ligands and metal ions, one‐pot crystallization condition would be optimized to make the crystallization of CMOFs based on the target anions faster than those of the CMOFs based on analogous anions, which can allow the target CMOFs to preferentially crystallize from the mixed‐anion solutions, thus further enhancing the selectivities for target anions. We anticipated that the cooperation between thermodynamical and kinetic control could endow the target CMOFs with both increased thermodynamic stabilities and higher crystallization rates, thus achieving the highly selective separation of target anions from their analogs. Moreover, as the target anions act as charge‐balancing anions and are free and uncoordinated in the cationic frameworks, the resultant CMOFs could be recyclable via anion exchange. However, to date, the cooperatively controlled crystallization for separation of organic species has not been reported.

## 2 Results and Discussions

**Figure 2:**
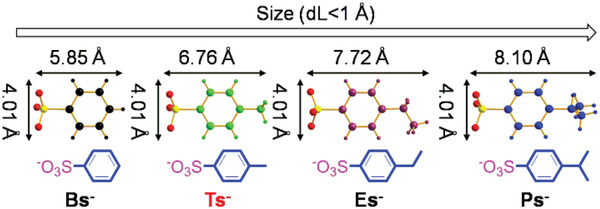
Structures of Tsˉ and its analogs. The black arrows denote the characteristic lengths of these anions.

To examine the viability of our strategy, we chose *p*‐toluenesulfonate anion (Tsˉ) as a typical anion as it is an important intermediate in the syntheses of dyes, medicine, pesticides, spices, and polymers.^[^ ^21^ ^]^ It is also used to improve the biological activity and solubility of active pharmaceutical salts.^[^ ^22^ ^]^ Moreover, the corresponding neutral molecule, *p*‐toluenesulfonic acid, is extensively used as a catalyst in various reactions such as 1,3‐dipolar cycloaddition, Friedlander–type cyclization, and Kabachnik–Field reaction.^[^ ^23^ ^]^ However, excess Tsˉ anions in water pose a large threat to environmental safety and human health due to their xenobiotic character.^[^ ^24^ ^]^ In addition to Tsˉ, industrial wastewater often contains its analogs such as benzenesulfonate (C_6_H_5_SO_3_ˉ, Bsˉ), *p*‐ethylbenzenesulfonate (*p*‐CH_3_CH_2_‐C_6_H_4_SO_3_ˉ, Esˉ) and *p*‐isopropylbenzenesulfonate [*p*‐(CH_3_)_2_CH‐C_6_H_4_SO_3_ˉ, Psˉ] (**Figure** **2**). The corresponding size difference (dL) is less than 1 Å (5.85 Å for Bsˉ, 7.72 Å for Esˉ, 8.10 Å for Psˉ vs 6.76 Å for Tsˉ). Furthermore, compared to the above analogs, Tsˉ has an intermediate size, and its separation from its analogs in wastewater therefore remains challenging.

### 2.1 Choosing Suitable Ligands and Metal Ions for Separation of Tsˉ

According to the structure of Tsˉ, 4,4′‐azo‐triazole (atrz) was strategically chosen as a neutral ligand as it is significantly longer (10.8 Å) than Tsˉ (Figure 4), and features good solubility and strong coordination ability in water, which facilitates the construction of a suitable cationic framework for the encapsulation of Tsˉ into its pore.^[^ ^25^ ^]^ It has been extensively used as a ligand for the construction of various CMOFs with fascinating topological structures in water.^[^ ^26^ ^]^ Moreover, it has several C—H bonds while Tsˉ contains a SO_3_ˉ group, which facilitates the formation of intermolecular interactions (e.g., hydrogen–bonding interactions) between atrz and Tsˉ.

**Figure 3:**
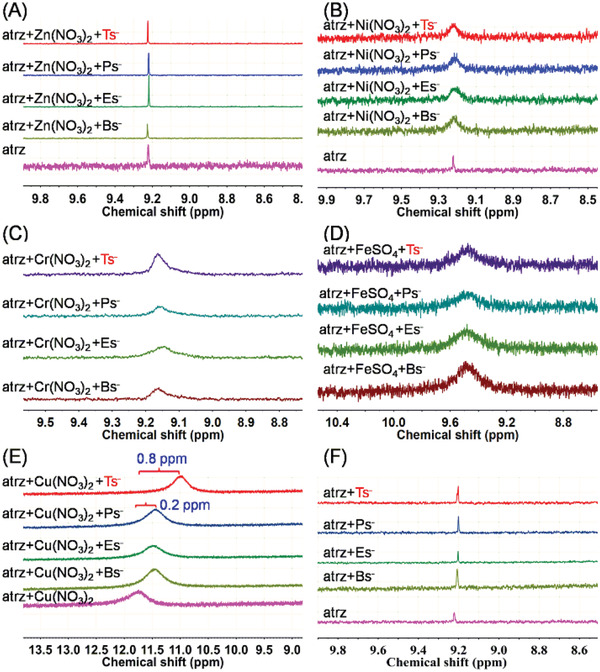
^1^H NMR spectra of various D_2_O solutions: A) atrz, Zn(NO_3_)_2_, and various sulfonate anions. B) atrz, Ni(NO_3_)_2_, and various sulfonate anions. C) atrz, Cr(NO_3_)_2_, and various sulfonate anions. D) atrz, FeSO_4_, and various sulfonate anions. E) atrz, Cu(NO_3_)_2_, and various sulfonate anions. F) atrz and various sulfonate anions. The peaks in all spectra are ascribed to atrz. Condition: metal ions (6.25 µmol), atrz (12.5 µmol), Tsˉ or its analogs (12.5 µmol), D_2_O (0.7 mL), RT.

Based on the chosen ligand, ^1^H NMR titration was used to investigate the interaction between various metal ions, the ligand, and Tsˉ or its analogs (Bsˉ, Esˉ, and Psˉ) to select a matched metal ion. Several commonly used metal ions (Zn^2+^, Ni^2+^, Cr^2+^, Fe^2+,^ and Cu^2+^) were chosen and separately added at the same concentration to a D_2_O solution containing atrz and Tsˉ or its analogs, and changes in the ^1^H NMR signal of atrz in the solution were recorded (**Figure** **3** and Figures S1–S6, Supporting Information). When Zn^2+^, Ni^2+^, Cr^2+^, and Fe^2+^ were separately added to a solution containing atrz and Tsˉ or its analogs, the signal of atrz in all the solutions maintained unchanged (Figure 3A–D), which demonstrated the interactions between the metal ions, atrz, and Tsˉ were identical to those between the metal ions, atrz, and the analogs. When the same amount of Cu^2+^ was added into the solution containing atrz and the analogs, the signals of atrz shifted upfield by ≈0.2 ppm (11.5 ppm) compared to that of atrz in a solution containing Cu^2+^ but no analogs (11.7 ppm, Figure 3E, pink curve). Surprisingly, when Cu^2+^ was added to the solution of atrz and Tsˉ, a dramatic change in the ^1^H NMR spectra was observed and the signal of atrz shifted upfield by 0.8 ppm (Figure 3E, red curve), which is four times longer than those for its analogs at the same condition. However, in the absence of Cu^2+^, the signal of atrz in the Tsˉ solution was consistent with those observed for the analogs (Figure 3F). These results demonstrated that the intermolecular interactions between Tsˉ, Cu^2+^, and atrz are possibly stronger than those observed for the analogs, which could contribute to the formation of a more stable CMOF and improve the selectivity for Tsˉ. Therefore, Cu^2+^ was chosen as a matched metal ion to construct the corresponding CMOF for the separation of Tsˉ from its analogs.

### 2.2 Optimization of Condition for CMOF Crystallization Rate Control

**Figure 4:**
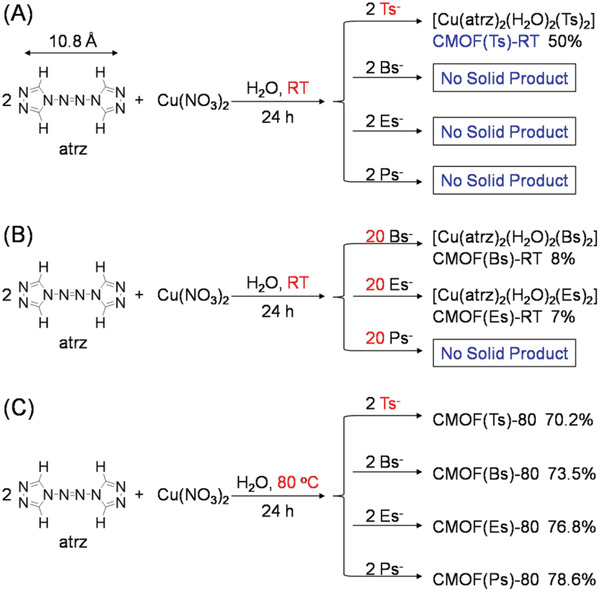
Synthesis of CMOFs based on Tsˉ and its analogs as charge‐balancing anions under various conditions: A) CMOF(Ts)‐RT under standard conditions; B) CMOF(Bs)‐RT and CMOF(Es)‐RT at RT; C) CMOF(Ts)‐80, CMOF(Bs)‐80, CMOF(Es)‐80, and CMOF(Ps)‐80 at 80 °C.

After the ligand and metal ion were chosen, Tsˉ and its analogs were separately added into their aqueous solutions to prepare the corresponding CMOFs [denoted as CMOF(X)‐T, where X = sulfonate anion and T = temperature]. In the proposed cooperatively controlled strategy, another key challenge is to find a suitable crystallization condition, under which the crystallization of the Tsˉ‐based CMOF is faster than those of analog‐based CMOFs. To achieve this purpose, we screened the crystallization conditions by varying the sulfonate anions/Cu^2+^ and atrz ligand ratio, temperature, and pH (see Supporting Information). When 2 equiv sulfonate anions were added into an aqueous solution of 2 equiv atrz and 1 equiv Cu(NO_3_)_2_ at pH = 5 and room temperature (RT), a great difference was observed between the crystallization rate of Tsˉ‐based CMOF and those of analog‐based CMOFs (**Figure** **4**A). In this set of experiments, these conditions were defined as standard.

**Figure 5:**
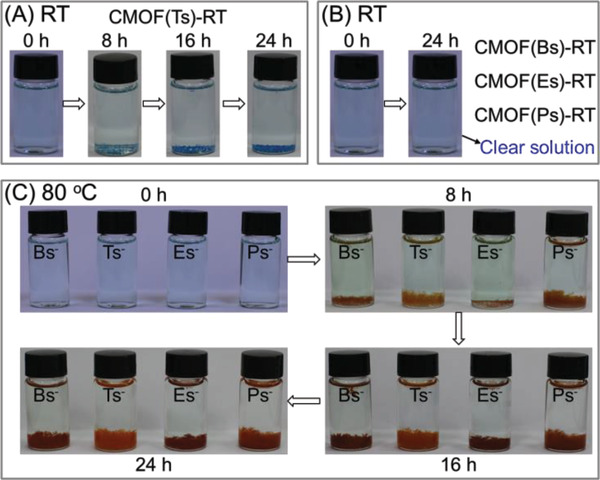
Crystallization process of various CMOFs based on Tsˉ and its analogs as charge‐balancing anions under various conditions: A) CMOF(Ts)‐RT under standard conditions; B) CMOF(Bs)‐RT, CMOF(Es)‐RT, and CMOF(Ps)‐RT under standard conditions; C) CMOF(Bs)‐80, CMOF(Ts)‐80, CMOF(Es)‐80, and CMOF(Ps)‐80 at 80 °C. Condition: sulfonate anions (0.25 mmol) were added into an aqueous solution (10 mL) containing atrz (0.25 mmol) and Cu(NO_3_)_2_ (0.125 mmol).

**Figure 6:**
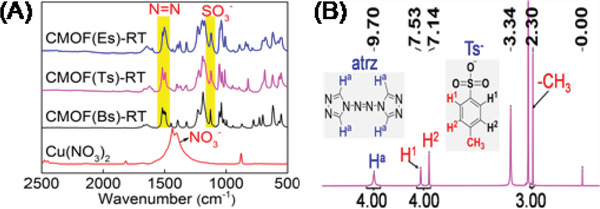
A) IR spectra of CMOF(Ts)‐RT, CMOF(Bs)‐RT, CMOF(Es)‐RT, and Cu(NO_3_)_2_. B) ^1^H‐NMR of CMOF(Ts)‐RT in DMSO‐*d*_6_.

Under standard conditions, the crystallization rate of the Tsˉ‐based CMOF was first investigated using a time‐course analysis of the amounts of solid products. Tsˉ (0.25 mmol) was added into an aqueous solution (10 mL) containing atrz (0.25 mmol) and Cu(NO_3_)_2_ (0.125 mmol) at RT. After 8 h, blue crystals (6.9 mg) [denoted as CMOF(Ts)‐RT] formed at the bottom of the solution (**Figure** **5**). The energy dispersive spectroscopy (EDS) elemental map (Figure S10, Supporting Information) showed that Cu, C, O, N, and S were evenly distributed within the resultant solid. Its infrared spectrum (**Figure** **6**A) showed that the appearance of the typical absorption peaks at ≈1190 cm^−1^ associated with SO_3_ group of Tsˉ and at 1550 cm^−1^ associated with N = N group of atrz, whereas the band of the NO_3_ˉ anion (1450 cm^−1^) was absent. Moreover, when the resultant solid was fully dissolved in [D_6_] DMSO, ^1^H NMR spectrum (Figure 6B) indicated that CMOF(Ts)‐RT contains Tsˉ anion [2.30 (‐CH_3_), 7.14 and 7.53 ppm] and the atrz ligand (9.70 ppm), and revealed the stoichiometry of Tsˉ and atrz to be 1:1. These results indicated that the solid crystals contained Cu ions, Tsˉ, and atrz, but not NO_3_ˉ, implying that this material could be a CMOF based on Tsˉ as a charge‐balancing anion.

**Figure 7:**
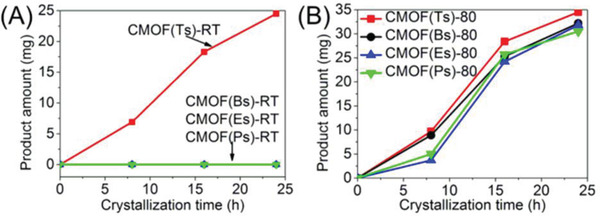
Time‐course analysis of the crystallization of CMOFs based on Tsˉ and its analogs as charge‐balancing anions at A) RT and B) 80 °C.

When the reaction time was extended to 16 h, the amount of crystals increased to 18.3 mg (Figures 5A and **7**A). The corresponding infrared (IR) and powder X‐ray diffraction (PXRD) spectra were identical to those of the products obtained at 8 h (Figures S14 and S15, Supporting Information). After 24 h, the product amount further increased to 24.5 mg while product structures remained unchanged. A further extension of the reaction time to 36 and 48 h did not affect the product amount or structure.

By contrast, when 2 equiv of the analogs such as Bsˉ, Esˉ, and Psˉ were separately added into the solution under the same condition (Figure 5B), no solid product was formed at the bottom of the solutions after 24 h and the solution remained clear even after 72 h. Their crystallization rates were almost zero (Figure 7A). Therefore, under standard conditions, CMOF(Ts)‐RT exhibited a remarkably faster crystallization than analog‐based CMOFs.

### 2.3 Intermolecular Interactions between Atrz Ligand and Sulfonate Anions

**Figure 8:**
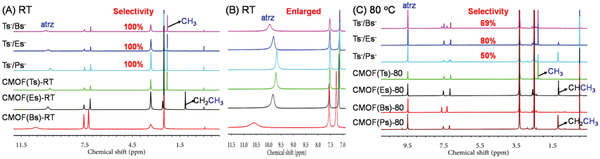
^1^H NMR spectra of various samples in *d_6_*‐DMSO: A) CMOF(Ts)‐RT, CMOF(Es)‐RT, CMOF(Bs)‐RT, and samples obtained from two‐component mixtures such as Tsˉ/Bsˉ, Tsˉ/Esˉ, and Tsˉ/Psˉ under standard conditions and B) their partial enlarged spectra. C) CMOF(Ts)‐80, CMOF(Es)‐80, CMOF(Bs)‐80, CMOF(Ps)‐80, and samples obtained from two‐component mixtures such as Tsˉ/Bsˉ, Tsˉ/Esˉ, and Tsˉ/Psˉ at 80 °C. Selectivity: the selectivity for Tsˉ.

To further confirm that the intermolecular interaction between Tsˉ, Cu^2+^, and atrz was stronger than those for the analogs, we attempted to obtain the corresponding single‐crystal CMOFs and used X‐ray crystallography to directly observe the differences of their molecular interactions. Under standard conditions, a number of CMOF(Ts)‐RT crystals were obtained, while analog‐based CMOFs could not be produced possibly because of low reactivity. In order to accelerate the reaction, the amount of added analogs was increased. When the amount of Bsˉ and Esˉ was increased from 2 to 20 equiv (Figure 4B), blue crystals [denoted as CMOF(Bs)‐RT and CMOF(Es)‐RT] were obtained after 24 h in yields of <10%. ^1^H NMR spectra (**Figure** **8**A,B; Figures S11 and S12, Supporting Information) indicated that CMOF(Bs)‐RT contains the Bsˉ anion (7.14 and 7.53 ppm) and atrz (9.70 ppm) while CMOF(Es)‐RT also contains the Esˉ anion (1.15, 7.13 and 7.53 ppm) and atrz (9.82 ppm). The ^1^H NMR spectra revealed that both the stoichiometries of the sulfonate anions and atrz were 1:1, as in the case of CMOF(Ts)‐RT. Despite numerous attempts, no crystal of CMOF(Ps)‐RT was obtained. These results further indicated that under standard conditions, CMOF(Ts)‐RT crystallizes easily, in contrast to analog‐based CMOFs.

**Figure 9:**
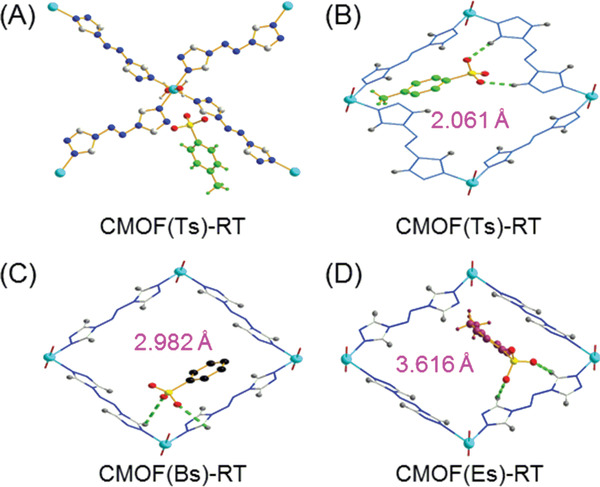
A) Coordination modes for CMOF(Ts)‐RT. B–D) Intermolecular hydrogen bonds (C—H∙∙∙O) in CMOF(Ts)‐RT, CMOF(Bs)‐RT, and CMOF(Es)‐RT with marked distance of the shortest hydrogen bond. Green dashed lines indicate intermolecular hydrogen bonds.

The as‐synthesized crystals [CMOF(Ts)‐RT, CMOF(Bs)‐RT, and MOF(Es)‐RT] were subjected to single‐crystal X‐ray diffraction analysis and were found to have the same asymmetry units, containing one Cu^II^ ion, two coordinated atrz units, two coordinated water molecules, and two sulfonate anions. In their structures, each Cu(II) atom also displays the same six‐coordinated octahedral geometry with a N_4_O_2_ donor set (**Figure** **9**A and Figures S16–S18, Supporting Information), bonding four different atrz molecules with four nitrogen‐coordinating sites, and the remaining two coordinating sites are occupied by two axially coordinated water molecules. Each atrz molecule acts as a bidentate ligand and bridges the adjacent Cu(II) atoms (Figure 9B–D), thereby extending to form a 2D infinite layer with the repeating [Cu_4_(atrz)_4_(H_2_O)_8_] unit in the ab plane. These layers further form the cationic frameworks with rhombus pores viewed along c axis. It is clearly observed that the sulfonate anions occupy the framework pores as free charge‐balancing anions.

The most significant difference between CMOF(Ts)‐RT, CMOF(Bs)‐RT, and CMOF(Es)‐RT was the length of the hydrogen bond (C‐H∙∙∙O) between the SO_3_^−^ groups of the sulfonate anions and the C‐H groups of atrz. In CMOF(Ts)‐RT, the distance ranged from 2.061 to 2.195 Å (Figure 9B), but was much longer in CMOF(Bs)‐RT (2.982–3.55 Å) and CMOF(Es)‐RT (3.616–4.246 Å) (Figure 9C,D). These results showed that CMOF(Ts)‐RT featured stronger intermolecular interactions between the cationic framework and the sulfonate anions than CMOF(Bs)‐RT and CMOF(Es)‐RT, in line with the results of ^1^H NMR titration.

To further confirm the strong intermolecular interaction in CMOF(Ts)‐RT, the binding energies of the cationic framework to Tsˉ and its analogs were calculated by first‐principle density functional theory (DFT), with the typical repeating cationic framework [Cu_4_(atrz)_4_(H_2_O)_8_] of CMOF(Ts)‐RT selected as a theoretical model. Figure S19, Supporting Information depicts the simulated structures of the corresponding CMOFs with Tsˉ and its analogs. As shown in **Table** **1**, the calculated binding energy of the cationic framework to Tsˉ (−254.4 kJ mol^−1^) was much higher than those for Bsˉ (−249.6 kJ mol^−1^), Esˉ (−245.4 kJ mol^−1^) and Psˉ (−249.1 kJ mol^−1^), which further confirmed that Tsˉ has stronger intermolecular interactions with the cationic framework than its analogs.

**Table 1:** DFT calculated data of CMOF(Bs)‐RT, CMOF(Ts)‐RT, CMOF(Es)‐RT, and CMOF(Ps)‐RT

Entry	*E*_f_ ^a)^ [hartree]	*E*_a_ ^b)^ [hartree]	*E*_c_ ^c)^ [hartree]	*E*_b_ ^d)^/kJ mol^−1^
CMOF(Bs)‐RT	−9548	−1710	−11 259	−249.6
CMOF(Ts)‐RT	−9548	−1788	−11 337	−254.4
CMOF(Es)‐RT	−9548	−1868	−11 416	−245.4
CMOF(Ps)‐RT	−9548	−1946	−11 495	−249.1

^a)^ Calculated energy of frameworks;

^b)^ Calculated energy of anion;

^c)^ Calculated energy of complex;

^d)^ Bing energy of the anion calculated by *E*_b_ = *E*_c −_ *E*_f −_ *E*_a_.

**Figure 10:**
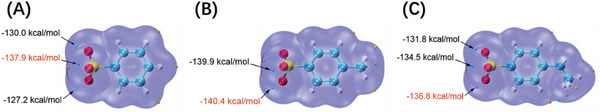
Electrostatic potential of A) Bsˉ anion, B) Tsˉ anion, and C) Esˉ anion calculated at B3LYP/6‐31++G(d, p) level. The electrostatic potentials are overall negative due to the charge of the anions and the local minima of ESP are plotted.

Interestingly, although Bsˉ, Tsˉ, and Esˉ all contain the same hydrogen‐bond acceptor group (SO_3_ˉ), why were the hydrogen‐bond interactions (intermolecular interactions) between the cationic frameworks and Tsˉ stronger than those for Bs^−^ and Es^−^? Electrostatic potential (ESP) was widely used to analyze the formation of non‐covalent interactions in the crystalline state, such as hydrogen bonds and halogen bonds.^[^ ^27^ ^]^ To demonstrate the differences of hydrogen‐bond interactions for three sulfonate anions, we performed their ESP calculations with in situ structures from the X‐ray crystal structures by Gaussian 09W program at the density function B3LYP/6‐31++g(d,p) level. As shown from **Figure** **10**, ESP map of Tsˉ was overall negative due to its charge and the surface local minimum was presented in the SO_3_ˉ group, which act as the hydrogen‐bond acceptor to form hydrogen‐bond interactions with the cationic frameworks. It was in agreement with the hydrogen‐bond interactions observed in the X‐ray crystal structure of CMOF(Ts)‐RT (Figure 9). In addition, the detailed surface analysis of Tsˉ calculated by ESP was summarized in Figure S55, Supporting Information. Meanwhile, the ESP maps with relative surface local minima of Bsˉ and Esˉ were also calculated and their surface local minima were also presented at the SO_3_ˉ group. The electron density is a common measure of the interaction energies in complexes bound by hydrogen bond.^[^ ^27c^ ^]^ A high electron density usually corresponds to a strong hydrogen bonding interaction. The ESP maps (Figure 10) showed that the surface local minimum of Ts^−^ was −140.4 kcal mol^−1^, which is remarkably lower than that of both Bs^−^ (−137.9 kcal mol^−1^) and Es^−^ (−136.8 kcal mol^−1^). The results indicated that the SO_3_^−^ group in Ts^−^ was the most electron‐rich one among the three sulfonate anions. Thus, Ts^−^ can form stronger hydrogen‐bond interaction with the cationic frameworks as hydrogen‐bond acceptor.

In addition, single crystal X‐ray diffraction indicated that the stoichiometry of Tsˉ and atrz in CMOF(Ts)‐RT with the formula {[Cu(atrz)_2_(H_2_O)_2_](Ts)_2_]} was 1:1, which agree with the result of ^1^H NMR measurements (Figure 6B). If NO_3_ˉ anions were co‐present inside the solid, the above stoichiometry of Tsˉ and atrz cannot be 1:1. Therefore, it also confirmed that the resultant crystal possesses an exceptionally high phase purity. More importantly, the above result indicated that the stoichiometric Tsˉ anions were encapsulated into the cationic framework pores and that all pores were completely occupied by Tsˉ anions, which is difficult to achieve for traditional porous materials. Possibly, the behavior can be ascribed to the need for the cationic framework to preserve charge balance by absorbing the stoichiometric Tsˉ anions into the pores via electrostatic interaction.

### 2.4 Thermodynamic Stability of CMOFs

**Figure 11:**
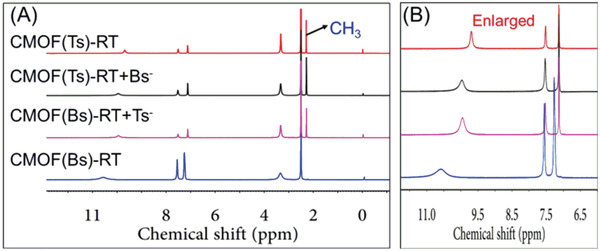
A) ^1^H NMR (solvent: *d_6_*‐DMSO) and B) partial enlarged spectra of CMOF(Bs)‐RT, CMOF(Ts)‐RT and samples obtained from the anion‐exchange experiments such as CMOF(Ts)‐RT + Bs^−^ and CMOF(Bs)‐RT + Ts^−^.

**Figure 12:**
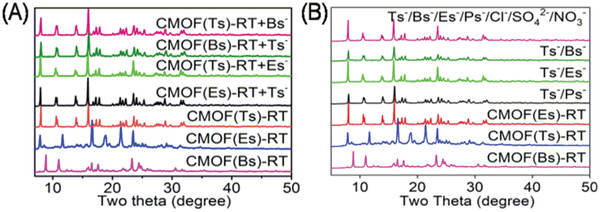
PXRD patterns of various samples: A) CMOF(Bs)‐RT, CMOF(Ts)‐RT, CMOF(Es)‐RT and samples obtained from anion‐exchange experiments such as CMOF(Ts)‐RT + Bsˉ, CMOF(Bs)‐RT + Tsˉ, CMOF(Ts)‐RT + Esˉ, and CMOF(Es)‐RT + Tsˉ. B) CMOF(Bs)‐RT, CMOF(Ts)‐RT, CMOF(Es)‐RT and samples obtained from selective experiments such as Tsˉ/Bsˉ, Tsˉ/Esˉ, Tsˉ/Psˉ, and Bsˉ/Tsˉ/Esˉ/Psˉ/Clˉ/SO_4_^2−^/NO_3_ˉ at RT.

**Figure 13:**
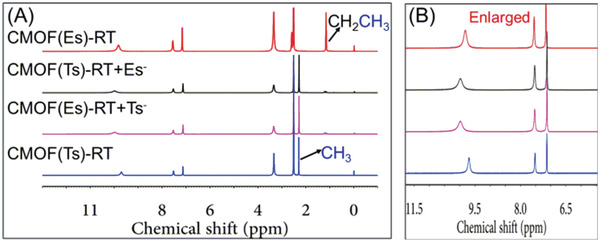
A) ^1^H NMR (solvent: *d_6_*‐DMSO) and B) partial enlarged spectra of CMOF(Es)‐RT, CMOF(Ts)‐RT and samples obtained from the anion‐exchange experiments such as CMOF(Ts)‐RT + Esˉ and CMOF(Es)‐RT + Tsˉ.

**Figure 14:**
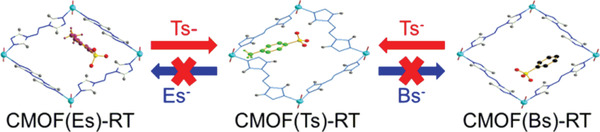
Schematic representation of the anion‐exchange reaction of CMOF(Es)‐RT, CMOF(Ts)‐RT, and CMOF(Bs)‐RT with Tsˉ, Bsˉ, and Esˉ.

Given that the intermolecular interactions between the cationic framework and Tsˉ were stronger than those for its analogs, the thermodynamic stabilities of the corresponding CMOFs were explored through a series of anion‐exchange experiments. As‐prepared CMOF(Bs)‐RT crystals were immersed into a fivefold molar excess of aqueous Na(Ts) at RT for 24 h, and the whole exchange process was followed visually and no crystal dissolution was observed. The resulting crystals were harvested and characterized by ^1^H NMR and PXRD. Its ^1^H NMR spectra (**Figure** **11** and Figure S20, Supporting Information) showed the characteristic signals of Tsˉ and atrz, while revealing the disappearance of the Bsˉ signal. The ^1^H NMR spectra also revealed the stoichiometry of Tsˉ and atrz to being 1:1, which is consistent with that of CMOF(Ts)‐RT. Meanwhile, its PXRD pattern was different from the precursor CMOF(Bs)‐RT, but identical to that of CMOF(Ts)‐RT (**Figure** **12**A). These results showed Bsˉ was fully substituted by Tsˉ and afford CMOF(Ts)‐RT. In contrast, when as‐prepared CMOF(Ts)‐RT crystals were immersed into a fivefold molar excess of aqueous Na(Bs) at RT for 24 h, it remained intact, as revealed by ^1^H NMR and PXRD (Figure 11 and Figure S21, Supporting Information). Even when the molar excess of Na(Bs) was increased to 83‐fold and the reaction time was extended to 72 h, CMOF(Ts)‐RT remained unchanged (Figure S24, Supporting Information). Moreover, when as‐prepared CMOF(Es)‐RT was immersed into a fivefold molar excess of aqueous Na(Ts) for 24 h, the ^1^H NMR spectrum and PXRD pattern of the formed crystals indicated that the occurrence of anion exchange to afford CMOF(Ts)‐RT (Figures 12A and **13**; Figure S22, Supporting Information). However, CMOF(Ts)‐RT remained intact in an 83‐fold molar excess of aqueous Na(Es) for 72 h (Figure S24, Supporting Information). Therefore, CMOF(Ts)‐RT was concluded to be more stable than CMOF(Bs)‐RT and CMOF(Es)‐RT (**Figure** **14**). As expected, Tsˉ has stronger intermolecular interaction with the cationic framework than its analogs, thus forming a more thermodynamically stable structure.

### 2.5 Separation of Ts^−^ from Smaller and Larger Analogs

**Figure 15:**
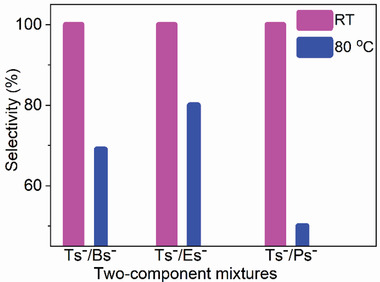
Selectivity for Tsˉ in two‐component mixtures such as Tsˉ/Bsˉ, Tsˉ/Esˉ, and Tsˉ/Psˉ at RT and 80 °C.

The faster crystallization and higher thermodynamic stability of CMOF(Ts)‐RT motivated us to examine competitive experiments between Tsˉ and its analogs under standard conditions for various two‐component mixtures of these sulfonate anions such as Tsˉ/Bsˉ, Tsˉ/Esˉ, and Tsˉ/Psˉ. Equimolar Tsˉ (0.25 mmol) and its smaller analog Bsˉ (0.25 mmol) were added into an aqueous solution (10 mL) containing atrz (0.25 mmol) and Cu(NO_3_)_2_ (0.125 mmol) at RT. After 24 h, a number of blue crystals, similar to CMOF(Ts)‐RT in color and shape, were obtained. The PXRD pattern and IR spectra of these crystals were identical to those of CMOF(Ts)‐RT (Figure 12B and Figure S27, Supporting Information). The corresponding ^1^H NMR spectrum(Figure 8A,B and Figure S28, Supporting Information) featured a series of characteristic signals from Tsˉ, and no signal of Bsˉ, while indicating the stoichiometry of Tsˉ and atrz to be still 1:1. If Bsˉ was co‐present inside the solid, the above stoichiometry of Tsˉ and atrz cannot be 1:1. These results showed that only Tsˉ anions entered the cationic framework of CMOF(Ts)‐RT with the selectivity of the cationic framework for Tsˉ being close to 100% (**Figure** **15**). In two additional two‐component experiments performed for its larger analogs Esˉ and Psˉ, the cationic framework also preferentially absorbed Tsˉ (Figures 8A,B and 15; Figures S29 and S30, Supporting Information). Moreover, Tsˉ/Esˉ was chosen as a model two‐component mixture and the crystallization process in the mixture was monitored over time by PXRD and IR spectroscopy (Figures S31 and S32, Supporting Information). The results showed that CMOF(Ts)‐RT was exclusively formed during the whole crystallization process, further confirming the high selectivity for Tsˉ. As expected, the cooperative effect of the faster crystallization and higher thermodynamic stability of CMOF(Ts)‐RT resulted in an extremely high selectivity for Tsˉ over its smaller and larger analogs. These results also confirmed the availability of our strategy for separation of intermediate‐size anionic pollutants from their analogs in water.

### 2.6 Selectivity for Ts^−^ under the Condition of Similar Crystallization Rate

To further demonstrate the significance of the cooperative control strategy, we explored another reaction condition, under which the crystallization rate of a Tsˉ‐based CMOF is almost equal to those of analog‐based CMOFs. The reactivities of organic ligands and metal ions as well as the crystallization rates of the corresponding CMOFs are well‐known to increase with increasing temperature. To facilitate comparison, the reaction temperature was increased to 80 °C while other reaction parameters were identical to those used under standard conditions. Specifically, 2 equiv Tsˉ or its analogs was separately added into an aqueous solution containing 2 equiv atrz and 1 equiv Cu(NO_3_)_2_ at 80 °C (Figure 4C). After 24 h, four kinds of orange crystals [denoted as CMOF(Ts)‐80, CMOF(Bs)‐80, CMOF(Es)‐80, and CMOF(Ps)‐80] were obtained (Figure 5C). The corresponding IR, PXRD, and ^1^H NMR spectra (Figures S33–S38, Supporting Information) consistently indicated that these crystals contained Cu cations, the sulfonate anions, and atrz, while the NO_3_ˉ anions were absent, which imply that these crystals could be CMOFs based on the sulfonate anions as charge‐balancing anions.

Crystallization rates were investigated in a similar manner as for CMOF(Ts)‐RT (Figure 5C). After 8 h, the amounts of the solids formed at the bottom of the solutions were 9.7 [CMOF(Ts)‐80], 8.9 [CMOF(Bs)‐80], 3.7 [CMOF(Es)‐80] and 5.0 mg [CMOF(Ps)‐80], respectively. IR and PXRD (Figures S39–S46, Supporting Information) showed that four solid products were CMOF(Ts)‐80, CMOF(Bs)‐80, CMOF(Es)‐80, and CMOF(Ps)‐80. When the reaction time was extended to 24 h, the amount of these crystals further increased to 34.5, 32.2, 31.8, and 30.5 mg and their structures remained unchanged. The above results suggested that their four products featured similar crystallization rates (Figure 7B), in contrast to the phenomenon observed under standard conditions.

The selectivities for Tsˉ under this condition were then investigated by testing various binary mixtures such as Tsˉ/Bsˉ, Tsˉ/Esˉ, and Tsˉ/Psˉ at 80 °C. Tsˉ (0.25 mmol) and Bsˉ (0.25 mmol) were added to an aqueous solution (10 mL) containing atrz (0.25 mmol) and Cu(NO_3_)_2_ (0.125 mmol) at 80 °C. After 24 h, a number of red solids were formed at the bottom of the solution. The ^1^H NMR spectra showed that the solids contained both Tsˉ and Bsˉ, and the selectivity for Tsˉ was determined to be less than 69% by integrating the NMR peaks (Figures 8C and 15; Figures S47–S50, Supporting Information), and was exceedingly lower than the value obtained under the standard condition. In two additional two‐component experiments, the selectivities for Tsˉ were also low (80% for Tsˉ/Esˉ and 50% for Tsˉ/Psˉ). Thus, the above results further indicated the importance of the cooperative control strategy for the highly selective separation of challenging anions from their analogs.

### 2.7 Practical Application

To evaluate the practical applicability of the proposed strategy, we explored the selective removal of Tsˉ from multicomponent mixtures containing competing organic analogs and inorganic anions commonly found in industrial wastewater. Under standard conditions, a solution containing equimolar amount of six anions [Bsˉ/Tsˉ/Esˉ/Psˉ/Cl^−^/SO_4_^2−^] (0.25 mmol)], including both smaller/larger analogs as well as inorganic anions, was added into an aqueous solution (10 mL) containing atrz (0.25 mmol) and Cu(NO_3_)_2_ (0.125 mmol). After 24 h, a number of blue crystals similar to CMOF(Ts)‐RT in color and shape were obtained. The related PXRD pattern and ^1^H NMR spectra showed that the solids were mostly CMOF(Ts)‐RT (Figure 12B and Figure S50, Supporting Information). The selectivity for Tsˉ in the mixtures containing more than seven competing anions [Bsˉ/Tsˉ/Esˉ/Psˉ/Cl^−^/SO_4_^2−^/NO_3_^−^] was determined to exceed 83% (Figure S50, Supporting Information), which indicate the simultaneous exclusion of smaller/larger analogs and inorganic anions.

### 2.8 Recovery and Reusability

**Figure 16:**
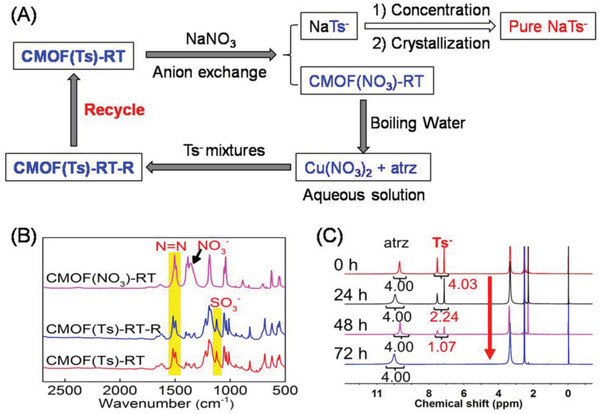
A) Overall Tsˉ separation cycle. B) IR spectra of CMOF(Ts)‐RT, MOF(NO_3_)‐RT, and CMOF(Ts)‐RT‐R. C) ^1^H NMR spectra (solvent: *d_6_*‐DMSO) of the solid sample after MOF(Ts) was immersed into an aqueous solution of NaNO_3_ (0.5 m) at different times.

In view of the importance of recovery and reusability for scale‐up or commercial application, we examined the recyclability of our strategy (**Figure** **16**). Given that charge‐balancing Tsˉ occupied the framework channels and were uncoordinated to the Cu centers in CMOF(Ts)‐RT, and that sufficiently large channels were available for anion excess, an anion exchange experiment was performed. As‐prepared CMOF(Ts)‐RT (100 mg) was immersed into a fivefold molar excess of aqueous NaNO_3_ at RT. After 72 h, a highly crystalline blue solid [denoted as CMOF(NO_3_)‐RT] was obtained. The related IR spectrum (Figure 15B) showed a strong band associated with the NO_3_^−^ anion (1450 cm^−1^), whereas the band of SO_3_ group of the Ts^−^anion (1450 cm^−1^) was absent, which agreed with ^1^H NMR data (Figure S51, Supporting Information). The anion‐exchange process was monitored over time. The resulting crystals were harvested at different times and characterized by ^1^H NMR analysis, which showed that the amount of Ts^−^anion present in the solid decreased with increasing exchange time (Figure 16C). These results revealed that anion‐exchange between CMOF(Ts)‐RT and NaNO_3_ had successfully taken place. The exchanged Tsˉ anions in the solution were concentrated and crystallized to afford a high‐purity sodium *p*‐toluenesulfonate.

The obtained CMOF(NO_3_)‐RT was dissolved in boiling water, and the solution was then cooled to RT and supplemented with two‐component mixture containing Tsˉ and Esˉ under standard conditions. After 24 h, a number of blue crystals [denoted as CMOF(Ts)‐RT‐R] were obtained and identified as CMOF(Ts)‐RT based on their IR spectrum and PXRD diffraction (Figure 16B and Figure S52, Supporting Information). The corresponding ^1^H NMR spectrum revealed the stoichiometry of Tsˉ and atrz in the solid was still 1:1, confirming high purity (Figure S53, Supporting Information). Therefore, the above results indicated that the resultant CMOF can be recycled. Moreover, as every cycle produced high‐purity CMOF(Ts)‐RT crystals, the recycling performances remained almost unchanged upon cycling.

## 3 Conclusion

An effective strategy, cooperatively (thermodynamically and kinetically) controlled crystallization for the highly selective separation of intermediate‐size organic anions from their analogs in water was developed. By applying this strategy for separation challenging Tsˉ from its smaller and larger analogs, atrz and Cu^2+^ were chosen as suitable neutral ligand and metal ion because ^1^H NMR titration revealed there was more than four times the length of the shifted signal of atrz due to the addition of Tsˉ compared to its analogs. With the ligand and metal ion in hand, we established suitable conditions, under which the crystallization of a CMOF based on Tsˉ was much faster than those based on its competing analogs. Meanwhile, the resultant Tsˉ‐based CMOF was more thermodynamically stable than analog‐based ones because of stronger intermolecular interaction between the cationic framework and Tsˉ compared with its analogs. Thus, in view of the high thermodynamic stability and faster crystallization, the cationic framework exhibited a high selectivity for Tsˉ in mixed‐anion solutions. Particularly, in the two‐component mixtures containing smaller or larger analogs, all the selectivities for Tsˉ were almost 100%. Moreover, the resultant CMOF was recyclable. In contrast, when the crystallization rate of the Tsˉ‐based CMOF was equal to those of analog‐based CMOF, the selectivities were very low (e.g., 50% for Tsˉ/Psˉ), which further demonstrates the significance of the proposed strategy. The present study provides new insights into the separation of anionic analogs through construction of CMOFs featuring higher stabilities and faster crystallization. Moreover, we anticipate that the proposed strategy would be useful for selective separation of challenging and functional anions such as pharmaceuticals, nulcleotides, and coenzymes from mixed‐anion solutions, in particular, which are otherwise difficult or unfeasible through traditional methods.

## Conflict of Interest

The authors declare no conflict of interest.

## Supporting information

Supporting Information
