# Supplementary material for: Highly Selective Separation Intermediate‐Size Anionic Pollutants from Smaller and Larger Analogs via Thermodynamically and Kinetically Cooperative‐Controlled Crystallization
Source: Adv Sci (Weinh). 2021 Feb 1;8(6):2003243. doi: 10.1002/advs.202003243 (PMC7967070; doi:10.1002/advs.202003243)
Supplement: Supplementary file 1 — Supporting Information [file ADVS-8-2003243-s001.pdf]

© 2020 Wiley-VCH GmbH

ADVANCED SCIENCE
Open Access

Supporting Information

for *Adv. Sci.,* DOI: 10.1002/advs.202003243

Highly Selective Separation Intermediate-Size Anionic Pollutants from Smaller and Larger Analogues via Thermodynamically and Kinetically Cooperative-Controlled Crystallization

Yongsheng Mi, Chaofeng Zhao, Shaomin Xue, Ning Ding, Yao Du, Hui Su, Shenghua Li, Siping Pang**

Supporting Information

Highly Selective Separation Intermediate-Size Anionic Pollutants from Smaller and Larger Analogues via Thermodynamically and Kinetically Cooperative-Controlled Crystallization

Yongsheng Mi, Chaofeng Zhao, Shaomin Xue, Ning Ding, Yao Du, Hui Su, Shenghua Li, Siping Pang**

Contents

1. **Materials and methods**
2. **Exploration of the interactions between various metal ions, atrzand sulfonate anions**
3. **Screening conditions for the preparation of CMOF(Ts)-RT**
4. **Preparation and structure characterizations of CMOFs obtained at room temperature**
5. **Crystallographic data and structures of CMOFs-RT**
6. **Calculation of the binding energy of the positive framework with different sulfonate anions**
7. **Exploring the thermodynamic stabilities of CMOFs through anionexchange**
8. **Competition experiments in two-component mixtures at room temperature**
9. **Preparation and structure characterizations of CMOFs obtained at 80°C**
10. **Competition experiments in two-component mixtures at 80 °C**
11. **Competition experiments in multicomponent mixtures at room temperature**
12. **Recovery and Reusability**
13. **Electrostatic potential (ESP) of sulfonate anions**

1. Materials and methods

4,4′-azo-1,2,4-triazole (Atrz) was prepared according to our previous work. All other materials were commercially available and used without further purification. All the chemicals and solvents are purchased from Sigma Aldrich and, unless otherwise indicated, used as received. Copper dinitrate trihydrate was purchased from Beijing Chemical Reagent Company without further purification.

All new compounds were characterized by IR spectroscopy, ^{1}H NMR and single-crystal X-ray diffraction analysis. IR spectrum was recorded on a Bruker Tensor 27 spectrophotometer with HTS-XT (KBr pellets). NMR spectroscopy was performed on a Bruker AVANCE III 600 instrument with Me_4Si as an internal standard. Powder X-ray diffraction (PXRD) patterns of the samples were analyzed with monochromatized Cu Kα (λ = 1.54178 Å) incident radiation by Bruker D8 Advance X-ray diffractometer operating at 40 kV voltage and 50 mA current. PXRD patterns were recorded from 5° to 80° (2θ) at 298 K. IR spectrum was recorded on a Bruker Tensor 27 spectrophotometer with HTS-XT (KBr pellets).

Crystals suitable for X-ray crystallographic analysis were obtained as mentioned above. The crystal structure was determined by a Rigaku RAXIS IP diffractometer and SHELXTL crystallographic software package of molecular structure. Data were collected by the ω scan technique. The structure was solved by direct methods with SHELXS-97 and expanded by using the Fourier technique. The non-hydrogen atoms were refined anisotropically. The hydrogen atom was determined with theoretical calculations and refined with an isotropic vibration factor.

2. Exploration of the interactions between various metal ions, atrzand sulfonate anions

The ^{1}H NMR titration experiments were carried out by separately adding6.25 μmol of different metal ions such as Ni^{2+}, Co^{2+}, Cr^{2+}, Fe^{2+} and Cu^{2+} into a D_2O solution (0.7 mL) containing 12.5 μmol atrz and 12.5 μmol Ts^-or itsanalogues.

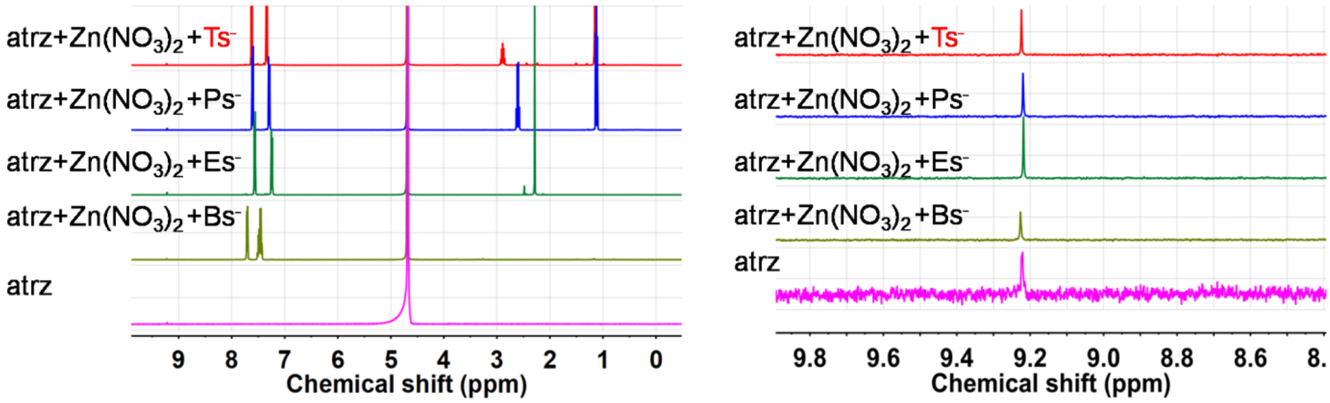

Figure S1. ^{1}HNMR spectra and partial enlarged spectra of the D_2O solution of atrz, $Zn(NO_3)_2$, and various sulfonate anions.

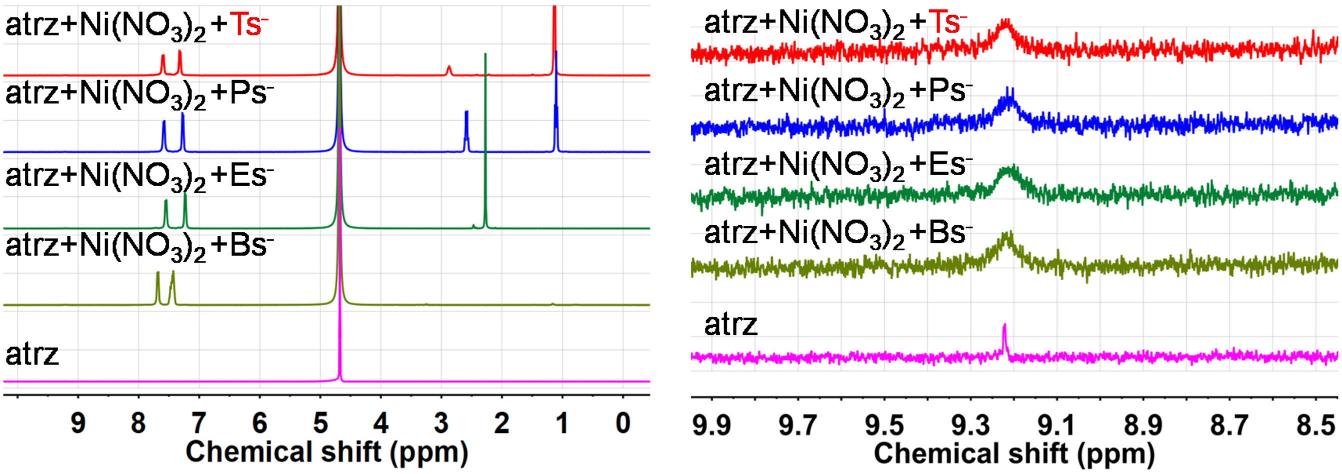

Figure S2. ^{1}HNMR spectra and partial enlarged spectra of the D_2O solution of atrz, $Ni(NO_3)_2$, and various sulfonate anions.

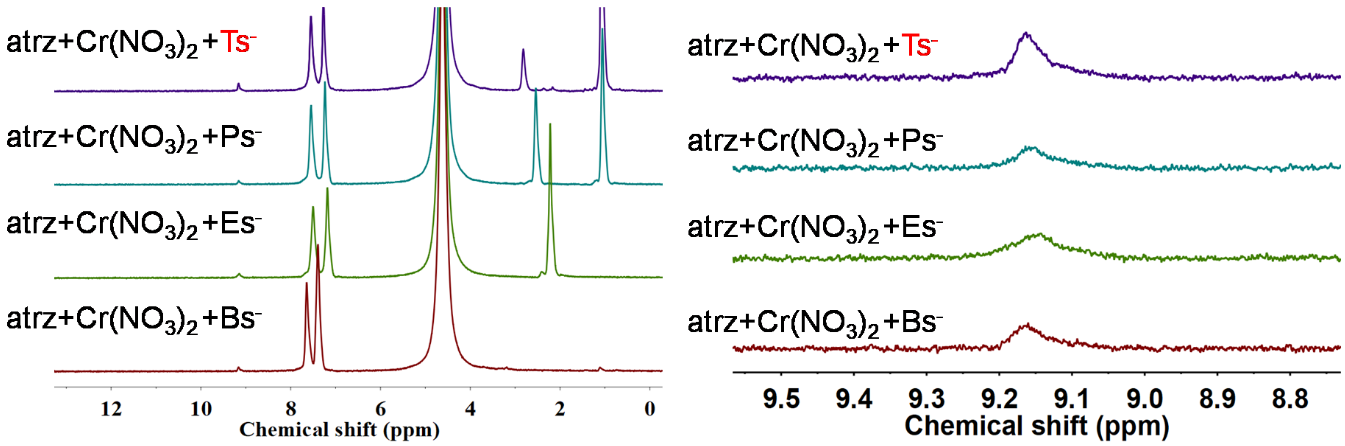

Figure S3. ^{1}HNMR spectra and partial enlarged spectra of the D_2O solution of atrz, $Cr(NO_3)_2$, and various sulfonate anions.

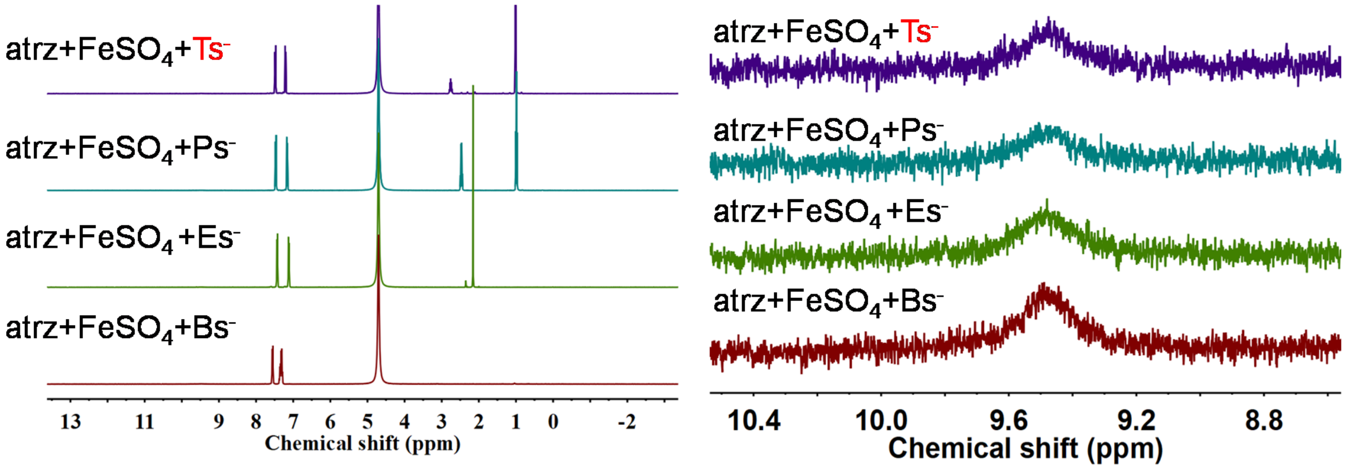

Figure S4. ^{1}HNMR spectra and partial enlarged spectra of the D_2O solution of atrz, $FeSO_4$, and various sulfonate anions.

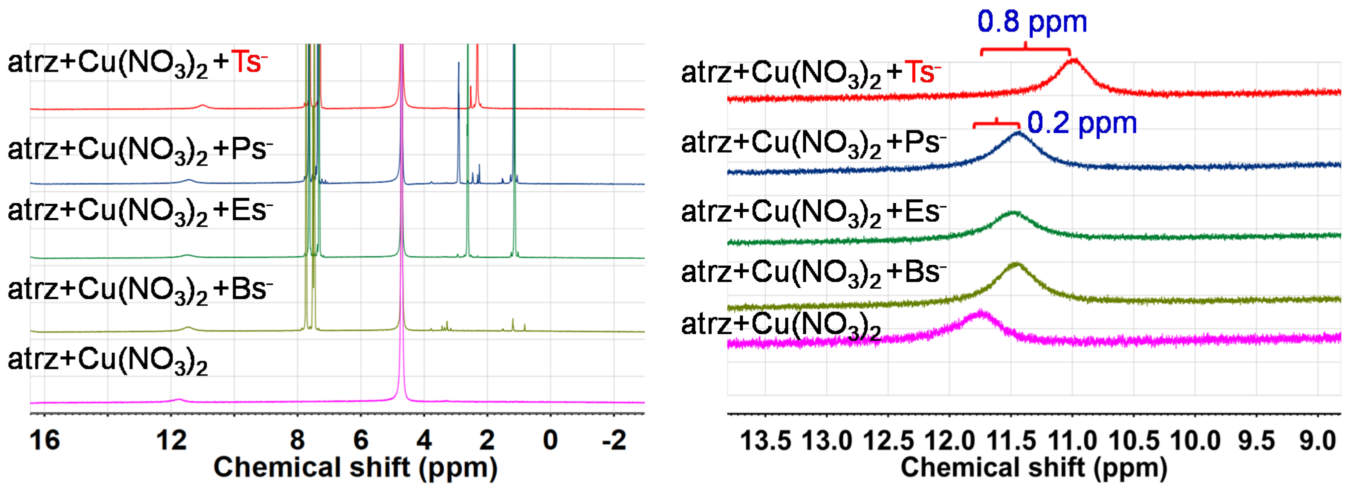

Figure S5. ^{1}HNMR spectra and partial enlarged spectra of the D_2O solution of atrz, $Cu(NO_3)_2$, and various sulfonate anions.

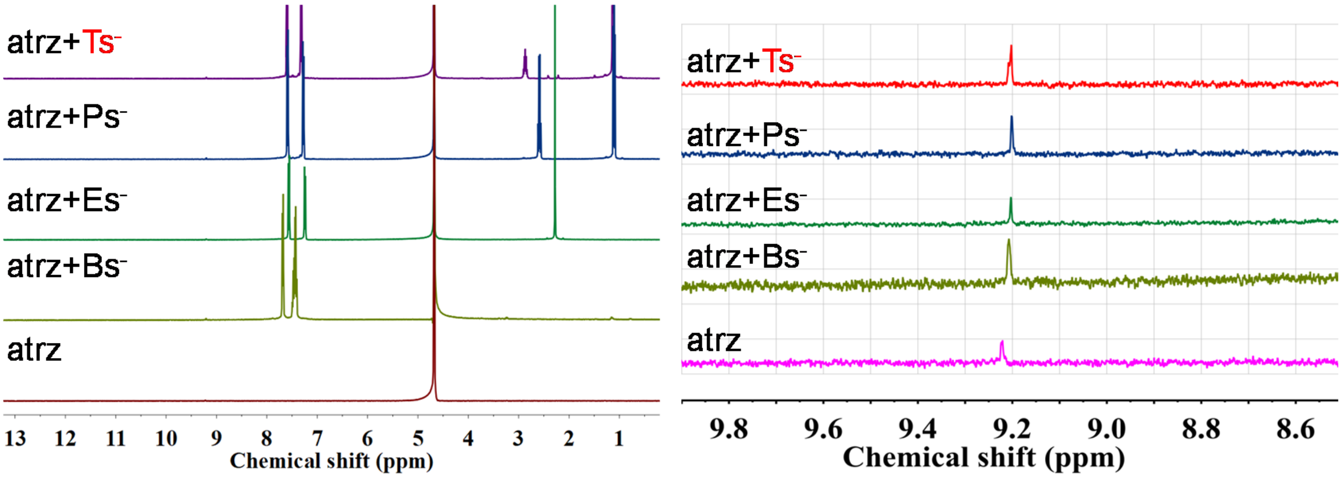

Figure S6. ^{1}HNMR spectra and partial enlarged spectra of the D_2O solution of atrzand various sulfonate anions.

3. Screening conditions for the preparation of CMOF(Ts)-RT

$Cu(NO_3)_2$, $AgNO_3$, and $Ni(NO_3)_2$ were separately added into aqueous solution containing atrz and benzenesulfonate anions at room temperature. For Ni^{2+}, the solutions containing Bs^-, Ts^- and Es^- kept clear after 24 h, while some light blue powder appeared in the solution containing Ps^-. Moreover, when Ag^+ was added into solution of all sulfonate anions, a large amount of solid powders immediately appeared. All the powder above had poor crystalline. Different with the above metal cations, when Cu^{2+} was added into aqueous solution containing atrz and Ts^-, a large amount of blue crystals (named CMOF(Ts)-RT) were produced at the bottom of the solution after 24 h, while solutions containing other sulfonate anions kept clear, which showed potential selectivity of Ts^- from its analogue anion. These results confirmed that Cu^{2+} was a matched metal ion.

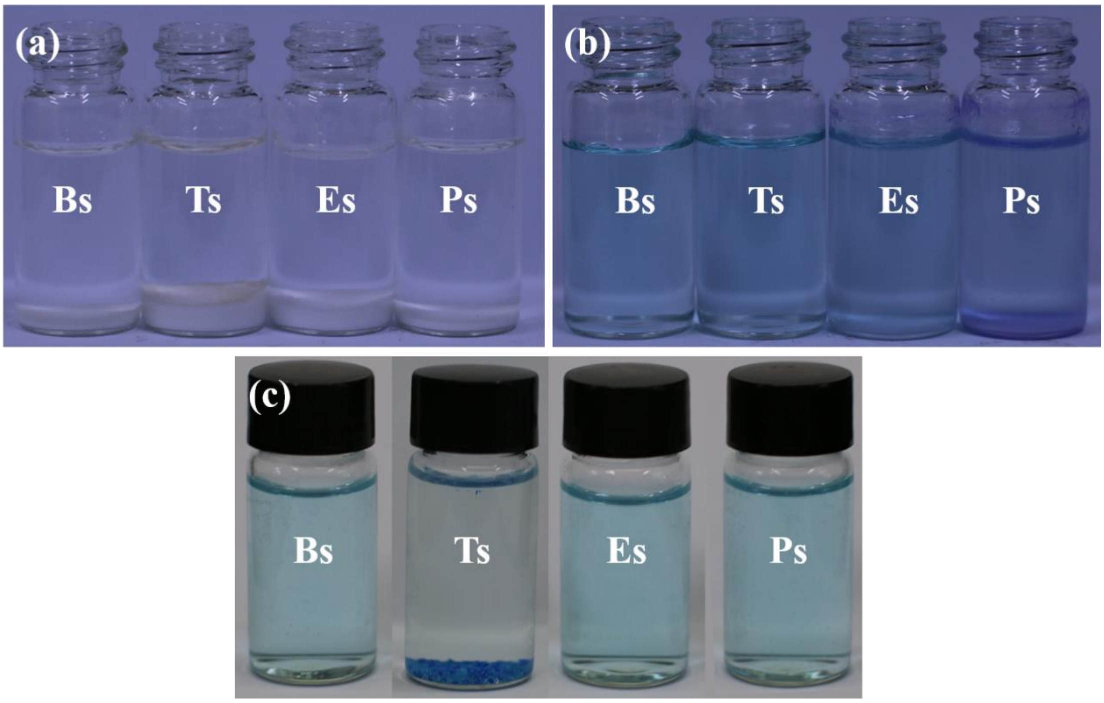

Figure S7. Screening of the preparation of CMOFs-RT using different metal ions such as Ag^{+} (a), Ni^{2+} (b) and Cu^{2+} (c).

At room temperature, the influence of atrz/$Cu(NO_3)_2$ /Ts^{-}ratio on preparation process was studied. After 24 h, the photographs for four reactions were taken and shown in Figure S8. When the ration of atrz/$Cu(NO_3)_2$ /Ts^{-} is 2:1:2, the yield is highest.

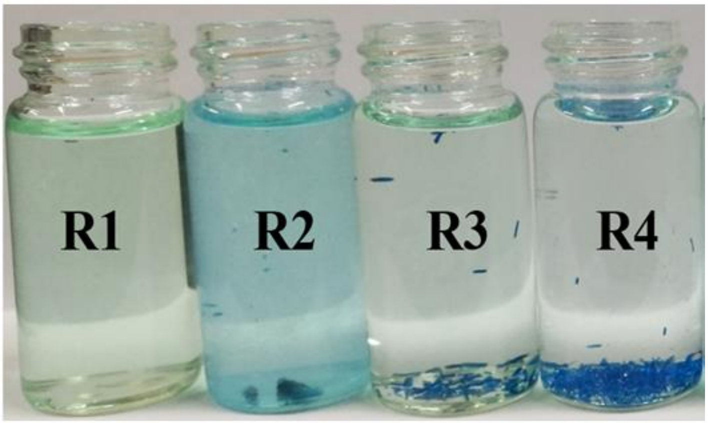

Figure S8. Optimization of the synthetic conditions of CMOF(Ts)-RT under different atrz/$Cu(NO_3)_2$ /Ts^{-} ratio: (R1) atrz/$Cu(NO_3)_2$ /Ts^{-}= 2: 0: 2 ; (R2) atrz/$Cu(NO_3)_2$ /Ts^{-}= 2:0.5: 2; (R3) atrz/$Cu(NO_3)_2$ /Ts^{-}= 2:2:2;

(R4) atrz/$Cu(NO_3)_2$ /Ts^{--}= 2:1:2.

Since the initial pH value of the solution was 5, the pH values of the solution were also screened by adding HCl or NaOH aqueous solution. As was shown in Figure S9, different pH values were adjusted and the results indicated that the initial pH value (pH = 5) was most suitable for the preparation of highly crystalline CMOF(Ts)-RT with a high yield. The effect of the reaction temperature was shown in the text.

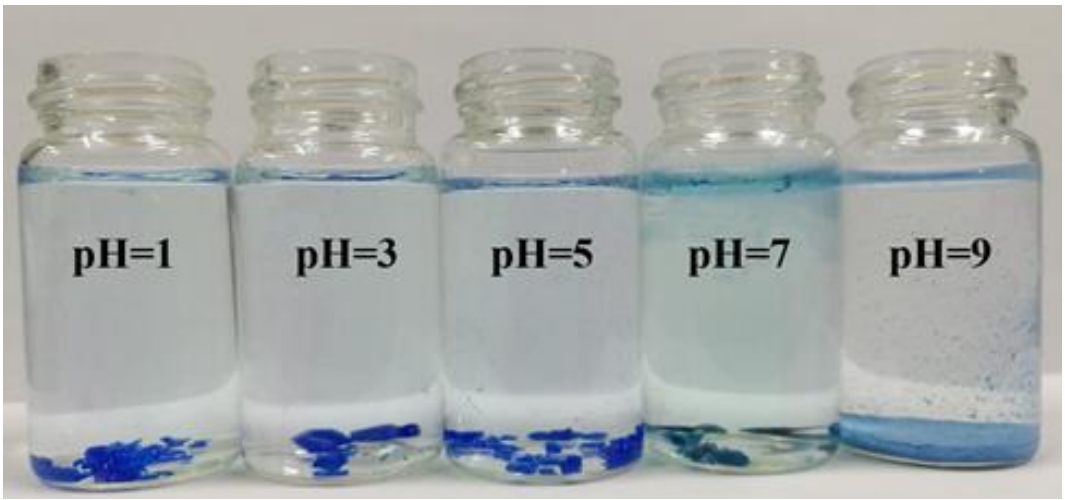

Figure S9. Optimization of the synthetic conditions of CMOF(Ts)-RT at different pH values. The ration of atrz/$Cu(NO_3)_2$ /Ts^{-} is 2:1:2

4. Preparation and structure characterizations of CMOFs obtained at room temperature

Preparation of CMOF(Ts)-RT: 0.125 mmol $Cu(NO_3)_2$ were added into a 10 mL aqueous solution containing 0.25 mmol atrz and 0.25 mmol sodium *p*-toluenesulfonate [Na(Ts)]. The resulting clear solution was placed under static condition at room temperature for 24 h, and then blue crystalline solids were produced at the bottom of the solution. After decanting the solution, the resultant crystals were washed thoroughly with deionized water, and dried in vacuum. The yield was ~50% based on atrz. IR (KBr pellets, λ, cm^{-1}): 3143, 1520, 1494, 1400, 1220, 1190, 1122, 1057, 1009, 820, 686, 618, 551. ^{1}H

NMR (600 MHz, DMSO-d_6): δ = 9.69 (s, 4H), 7.52 (d, J = 6.0 Hz, 2H), 7.13 (d, J = 6.0 Hz, 2H), 2.30 (s, 3H).

Preparation of CMOF(Bs)-RT: 0.125 mmol $Cu(NO_3)_2$ were added into a 10 mL aqueous solution containing 0.25 mmol atrz and 2.5 mmol sodium benzenesulfonate [Na(Bs)]. The resulting clear solution was placed under static condition at room temperature for 24 h, and then blue crystalline solids were produced at the bottom of the solution. After decanting the solution, the resultant crystals were washed thoroughly with deionized water, and dried in vacuum. The yield was ~ 8% based on atrz. IR (KBr pellets, λ, cm^{-1}): 3148, 1518, 1394, 1320, 1224, 1188, 1129, 1053, 1040, 775, 729, 701, 618, 553. ^1H NMR (600 MHz, DMSO-d_6): δ = 10.56 (s, 4H), 7.56 (s, 2H), 7.27 - 7.24 (m, 3H).

Preparation of CMOF(Es)-RT: 0.125 mmol $Cu(NO_3)_2$ were added into a 10 mL aqueous solution containing 0.25 mmol atrz and 2.5 mmol sodium 4-ethylbenzenesulfonate [Na(Es)]. The resulting clear solution was placed under static condition at room temperature for 24 h, and then blue crystalline solids were produced at the bottom of the solution. After decanting the solution, the resultant crystals were washed thoroughly with deionized water, and dried in vacuum. The yield was ~7% based on atrz. IR (KBr pellets, λ, cm^{-1}): 3119, 1518, 1500, 1379, 1324, 1231, 1196, 1172, 1120, 1040, 1005, 890, 840, 681, 620, 553. ^1H NMR (600 MHz, DMSO-d_6): δ = 9.81 (s, 4H), 7.55 (d, J = 6.0 Hz, 2H), 7.16 (d, J = 6.0 Hz, 2H), 2.60 – 2.58 (m, 2H), 1.16 – 1.14 (m, 3H).

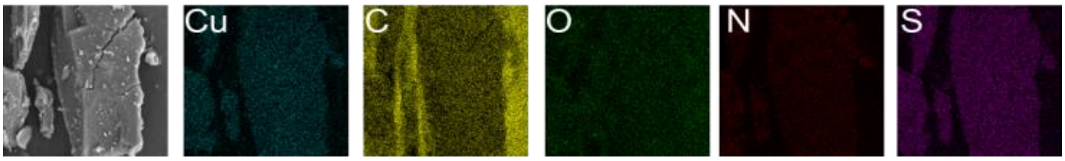

Figure S10. SEM-EDS mapping images of CMOF(Ts)-RT.

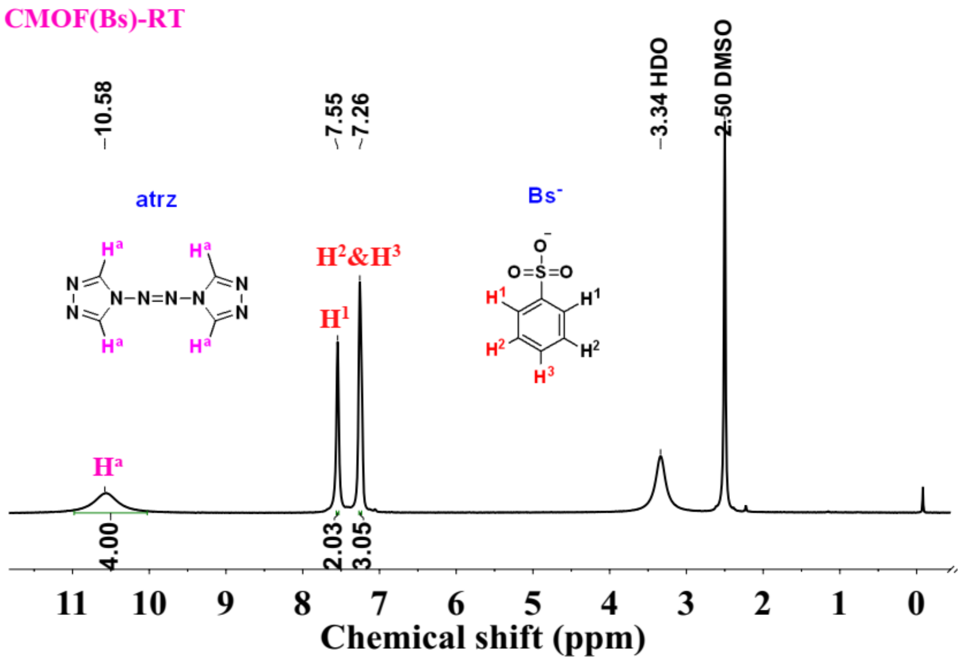

Figure S11. ^{1}H-NMR spectrum of CMOF(Bs)-RT dissolved in DMSO-d_6.

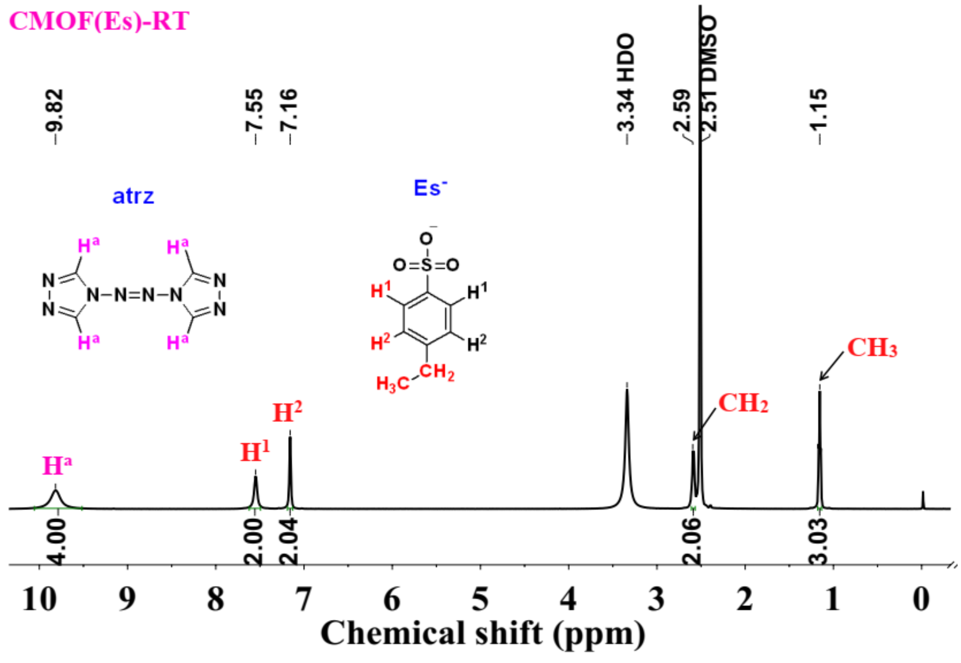

Figure S12. ^{1}H-NMR spectrum of CMOF(Es)-RT dissolved in DMSO-d_6.

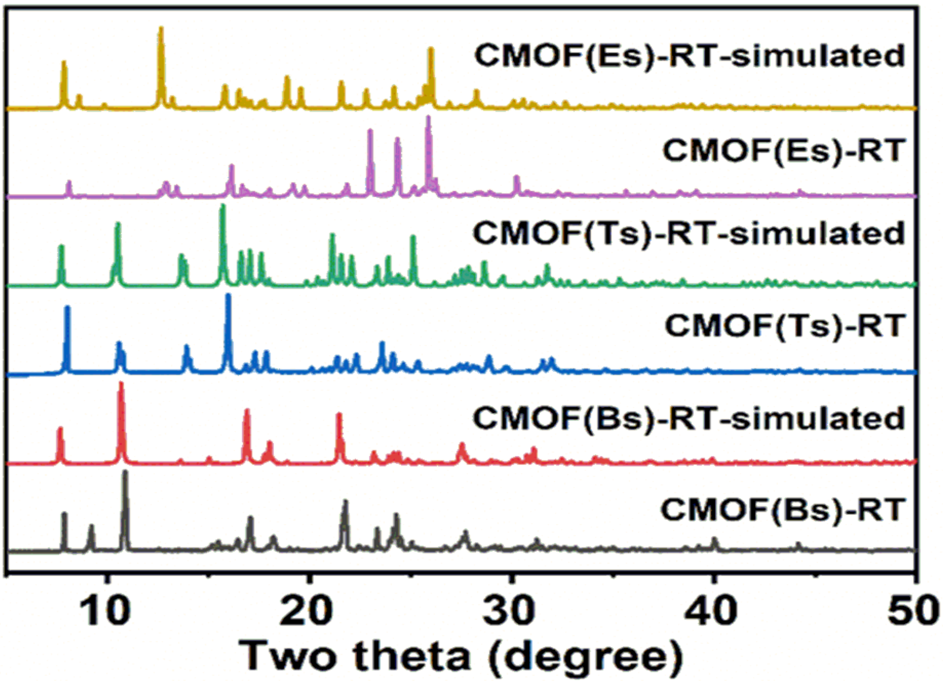

Figure S13. PXRD patterns of CMOF(Ts)-RT, CMOF(Bs)-RT, CMOF(Es)-RT and the simulated patterns

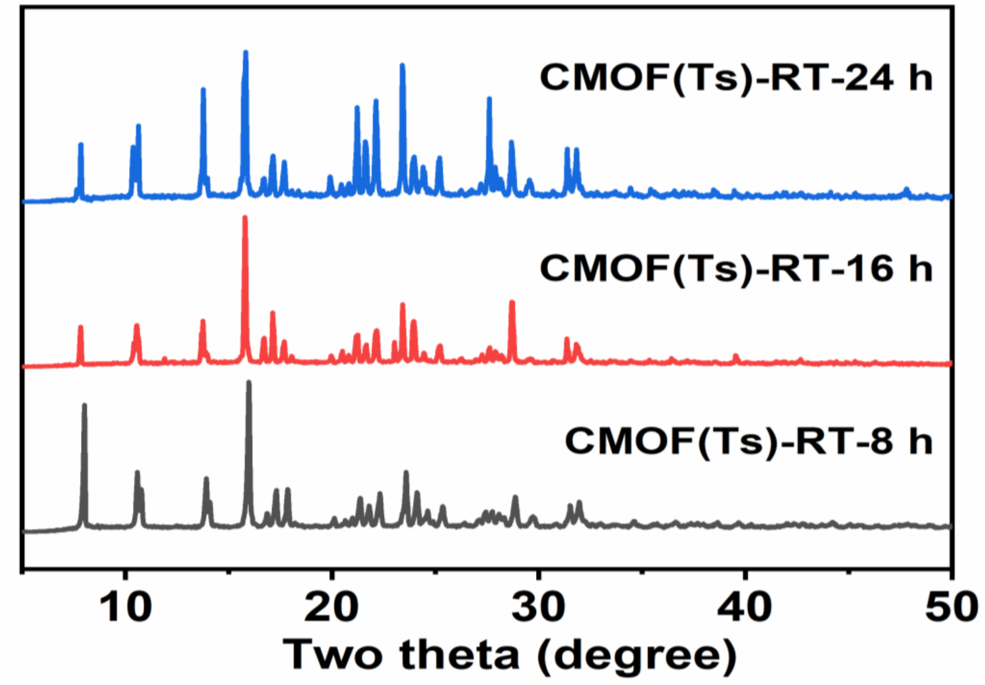

Figure S14. PXRD patterns of CMOF(Ts)-RT and the samples obtained from the reaction of 0.25 mmol Ts^-, 0.25 mmol atrz and 0.125 mmol $Cu(NO_3)_2$at different reaction times at room temperature.

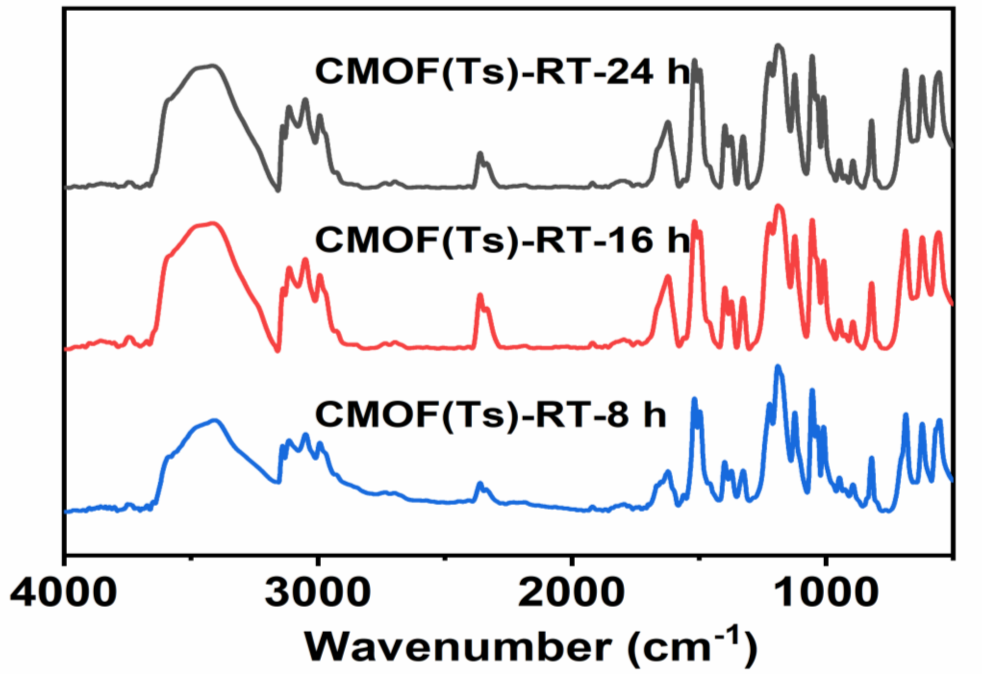

Figure S15. IR spectra of CMOF(Ts)-RT and the samples obtained from the reaction of 0.25 mmol Ts^-, 0.25 mmol atrz and 0.125 mmol $Cu(NO_3)_2$at different reaction times at room temperature.

5. Crystallographic data and structures of CMOFs-RT.

Table S1 Crystallographic data and structural refinement parameters of CMOF(Ts)-RT, CMOF(Bs)-RT and CMOF(Es)-RT.

**Crystal**	**CMOF(Bs)-RT**	**CMOF(Ts)-RT**	**CMOF(Es)-RT**
Formula	$C_{20}H_{26}CuN_{16}\ O_{10}\ S_2$	$C_{22}H_{30}CuN_{16}\ O_{10}\ S_2$	$C_{24}H_{38}CuN_{16}\ O_{12}\ S_2$
M_r (g/mol)	778.23	806.28	870.36
Crystal system	Monoclinic	Monoclinic	Triclinic
Space group, Z	*P*2$_1$/*c*, 2	*P*2$_1$/*c*, 2	*P*-1, 2
a (Å)	11.561(5)	11.588(2)	7.621(5)
b (Å)	11.778(5)	12.953(3)	11.165(7)
c (Å)	11.685(5)	11.200(2)	12.116(8)
α (°)	90	90	101.174(7)
β (°)	95.835(5)	99.570(2)	104.509(8)

γ (°)	90	90	106.351(7)
V (Å^3)	1582.8(12)	1657.6(6)	918.1(10)
D_c *(g/cm^3)*	1.633	1.615	1.574
temperature (K)	296	296	296
F(000)	798.0	830.0	451.0
μ (mm^{-1})	0.901	0.863	0.789
$2\theta_{max}/^o$	55.070	50.158	54.786
R_1, $wR_2[I>2\sigma(I)]$	0.0435, 0.1448	0.0517, 0.1691	0.0498, 0.1556
R_1, wR_2*(all data)*	0.0552	0.0598	0.0598
S on F^2	1.053	1.031	1.077

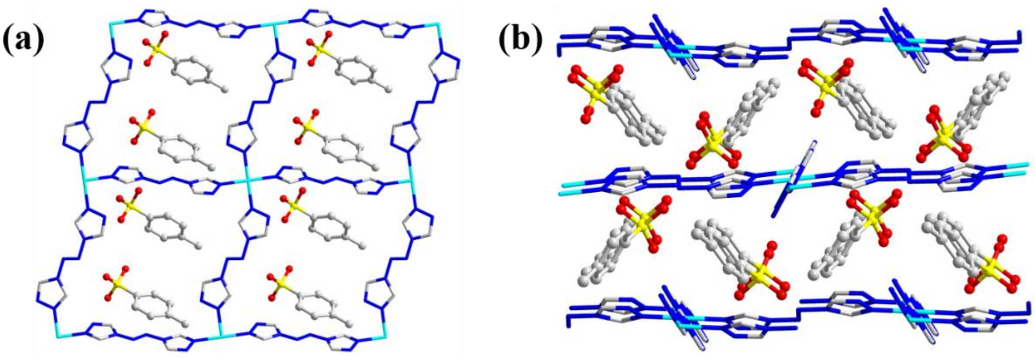

Figure S16. Packing structures of CMOF(Ts)-RT view along the crystallographic (a) b axis and (b) a axis. Hydrogen atoms and guest water have been omitted for clarity. Atom color: cyan, Cu; blue, N; red, O; gray, C; yellow, S.

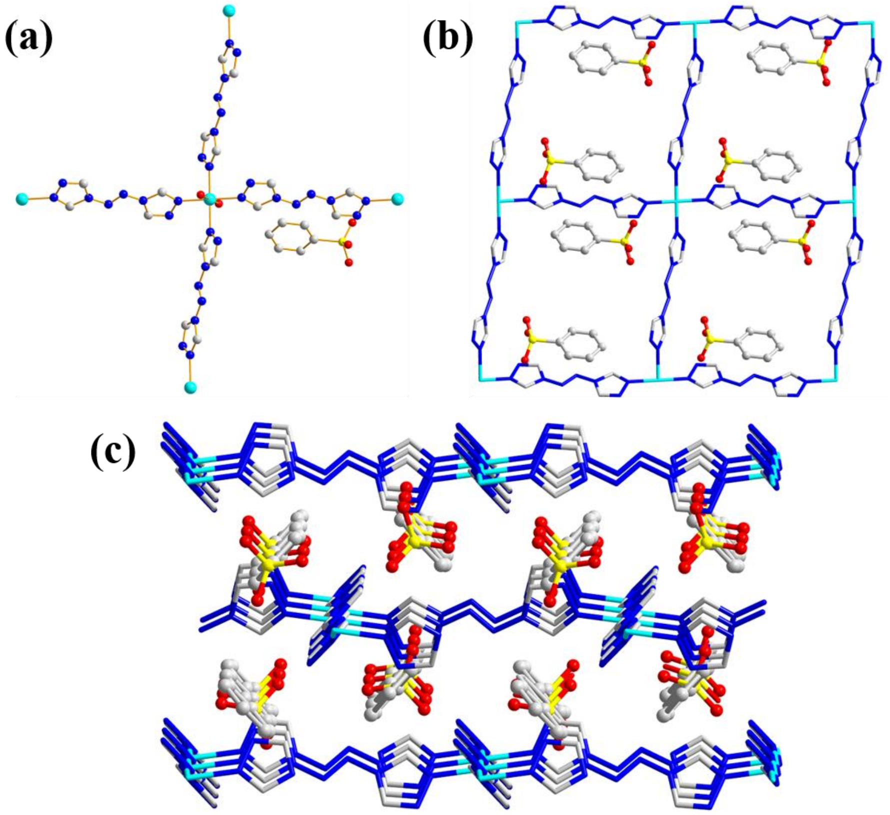

Figure S17. (a) Coordination structure and (b), (c) Packing structures of CMOF(Bs)-RT view along the crystallographic b axis and a axis, respectively. Hydrogen atoms and guest water have been omitted for clarity. Atom color: cyan, Cu; blue, N; red, O; gray, C; yellow, S.

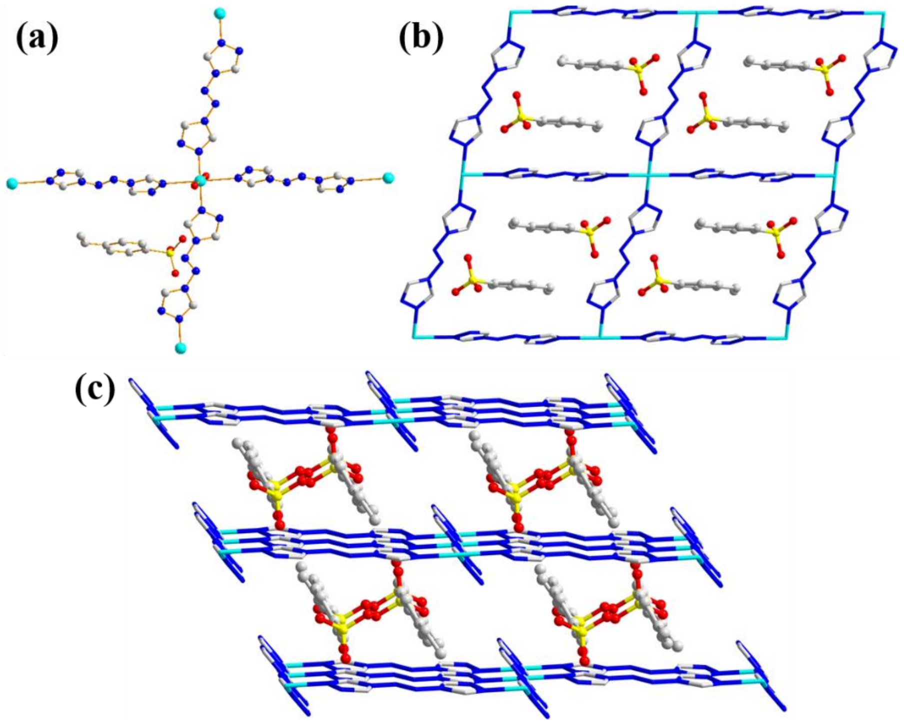

Figure S18. (a) Coordination structure and (b), (c) Packing structures of CMOF(Es)-RT view along the crystallographic a axis and c axis, respectively. Hydrogen atoms and guest water have been omitted for clarity. Atom color: cyan, Cu; blue, N; red, O; gray, C; yellow, S.

6. Calculation of the binding energy of the positive framework with different sulfonate anions

First-principle calculations based on density functional theory (DFT) B3LYP / GFN2-xTB methods were performed using the Gaussian 09 program. Fragments of CMOFs extracted from the experimentally obtained crystal structures were chosen as the computational models. Geometry optimizations were performed at the M062X-D3/SDD−6-31G(d, p) level, in which the M062X functional combined with the D3 version of Grimme's dispersion were employed, and the Stuttgart/Dresden relativistic effective core potentials (ECPs) with corresponding valence basis set (SDD) and the standard Gaussian-type 6-31G(d, p) basis set were applied for heavy atom (Cu) and light atoms (C, H, O and N), respectively. The C, N, O, and Cu atoms of CMOFs frameworks were freezed during optimizations. Single-point energies were calculated for the complexes, frameworks, and anions

after geometry optimizations at the same level, and the binding energies (E_b) between anions and frameworks were calculated by $E_b = E_{(complex)} - E_{(framework)} - E_{(anion)}$.

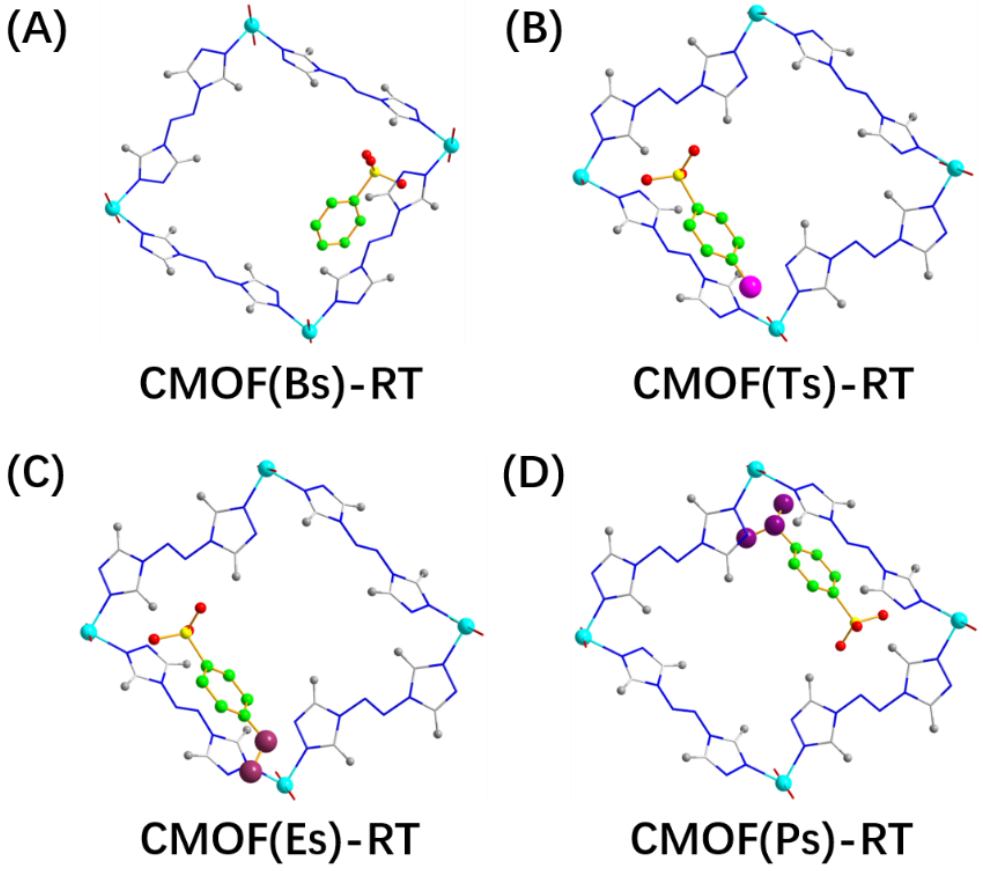

Figure S19. Simulated structures of the positive framework with different sulfonate anions optimized by DFT: (a) CMOF(Bs)-RT, (b) CMOF(Ts)-RT, (c) CMOF(Es)-RT and (d) CMOF(Ps)-RT.

7. Exploring the thermodynamic stabilities of CMOFs through anionexchange.

(1) Anion-exchange experiments between Ts^- and CMOF(Bs)-RT

39 mg (0.05 mmol) CMOF(Bs)-RT was immersed into 5 mL aqueous solution of sodium Ts^- (c = 0.05 mol L^{-1}). Blue crystals remained in the solution and was filtered after 24 h. The resulting solid was washed thoroughly with deionized water, dried in vaccum and named CMOF(Bs)-RT+Ts^-.

(2) Anion-exchange experiments between Bs^- and CMOF(Ts)-RT

Similarly, 25 mg (0.03 mmol) CMOF(Ts)-RT was immersed into 5 mL aqueous solution of sodium Bs^- (c = 0.5 mol L^{-1}). Blue crystals remained in the solution and was filtered after 3 days. The resulting solid was washed thoroughly with deionized water, dried in vaccum and named CMOF(Ts)-RT+Bs^-.

(3) Anion-exchange experiment between Ts^- and CMOF(Es)-RT

44 mg (0.05 mmol) CMOF(Es)-RT was immersed into 5 mL aqueous solution of sodium Ts^- (c = 0.05 mol L^{-1}). Blue crystals remained in the solution and was filtered after 24 h. The resulting solid was washed thoroughly with deionized water, dried in vaccum and named CMOF(Es)-RT+Ts^-.

(4) Anion-exchange experiment between Es^- and CMOF(Ts)-RT

Similarly, 25 mg (0.03 mmol) CMOF(Ts)-RT was immersed into 5 mL aqueous solution of sodium Es^- (c = 0.5 mol L^{-1}). Blue crystals remained in the solution and was filtered after 3 days. The resulting solid was washed thoroughly with deionized water, dried in vaccum and named CMOF(Ts)-RT+Es^-.

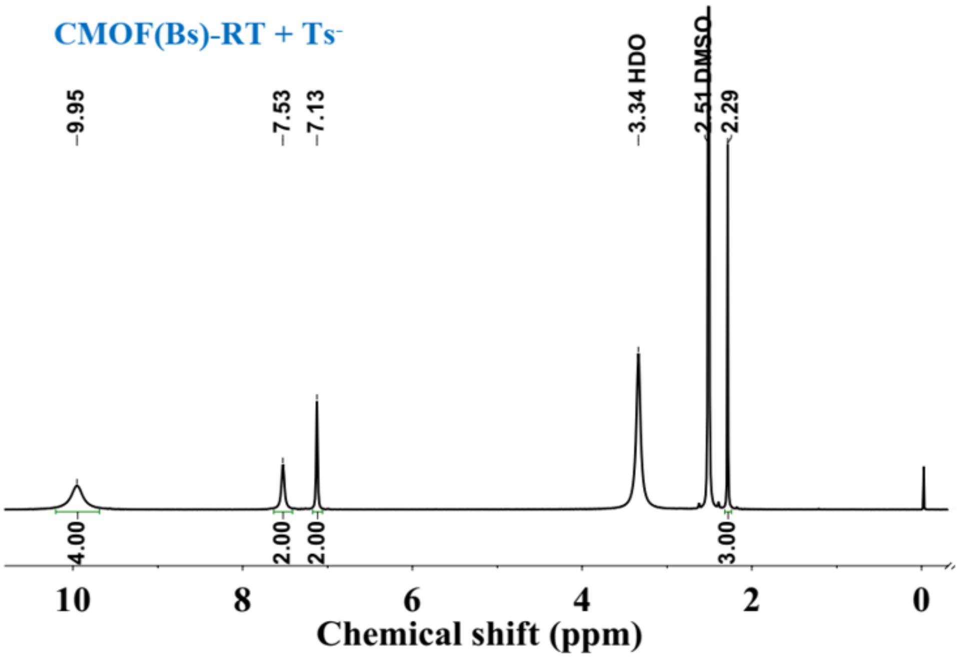

Figure S20. ^{1}H-NMR spectrum of the sample (dissolved in DMSO-d_6) obtained from the anion-exchange experiment between CMOF(Bs)-RT + Ts^- at room temperature.

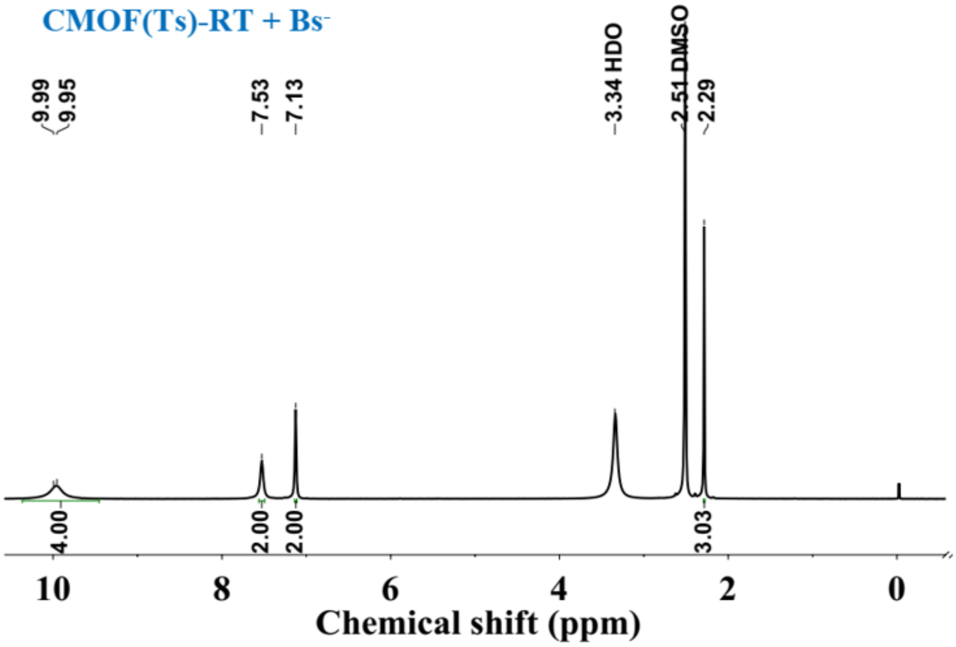

Figure S21. ^{1}H-NMR spectrum of the sample (dissolved in DMSO-d_6) obtained from the anion-exchange experiment between CMOF(Ts)-RT + Bs$^-$ at room temperature.

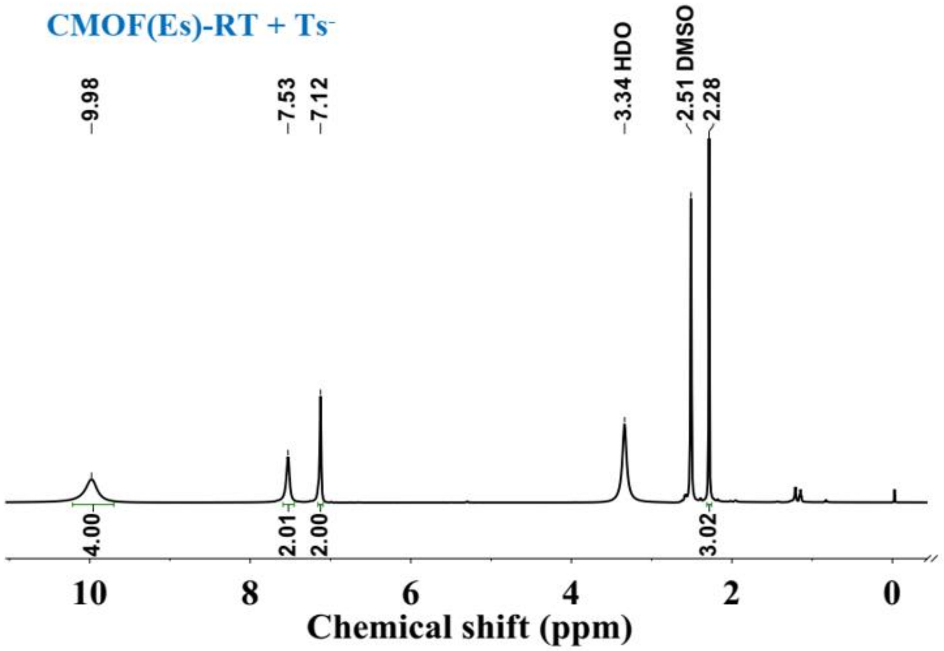

Figure S22. ^{1}H-NMR spectrum of the sample (dissolved in DMSO-d_6) obtained from the anion-exchange experiment between CMOF(Es)-RT + Ts$^-$ at room temperature.

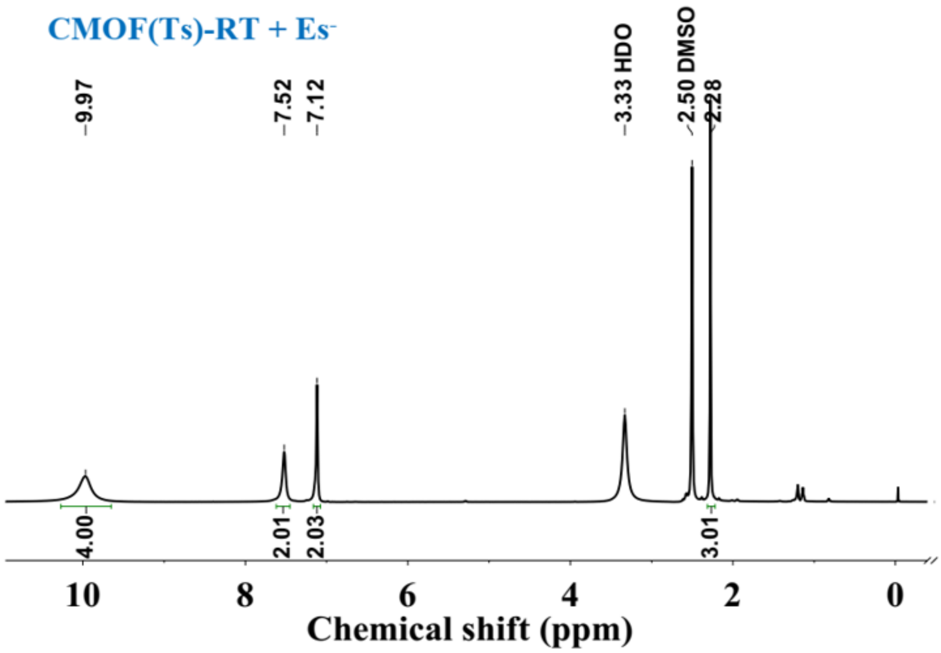

Figure S23. ^{1}H-NMR spectrum of the sample (dissolved in DMSO-d_6) obtained from the anion-exchange experiment between CMOF(Ts)-RT + Es^- at room temperature.

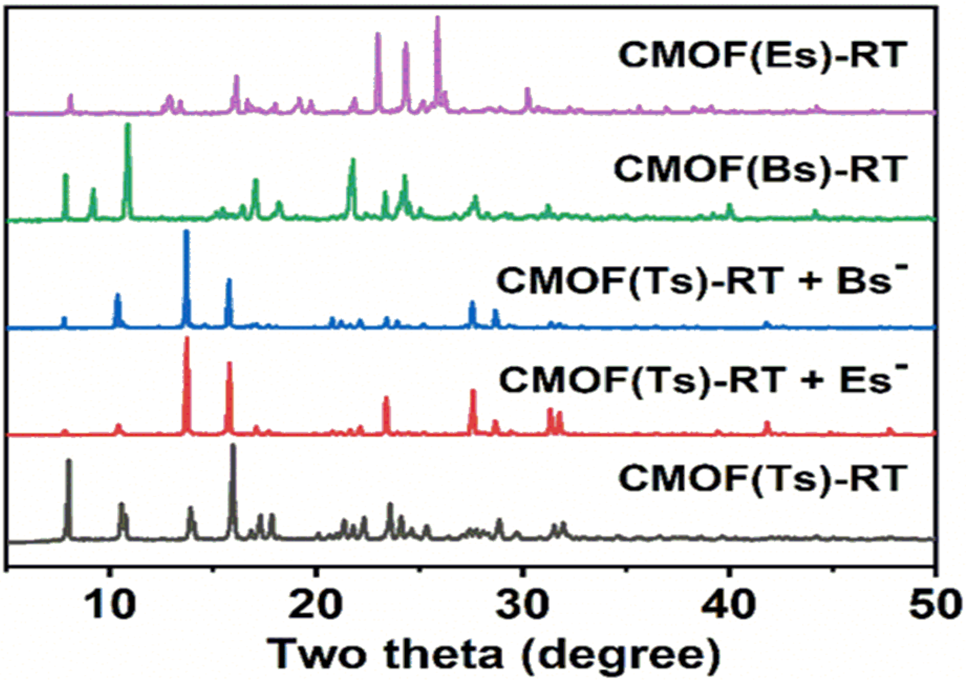

Figure S24. PXRD patterns of samples obtained from the anion-exchange experiments of CMOF(Ts)-RT immersed in 83-fold molar excess of aqueous Bs-& Es-.

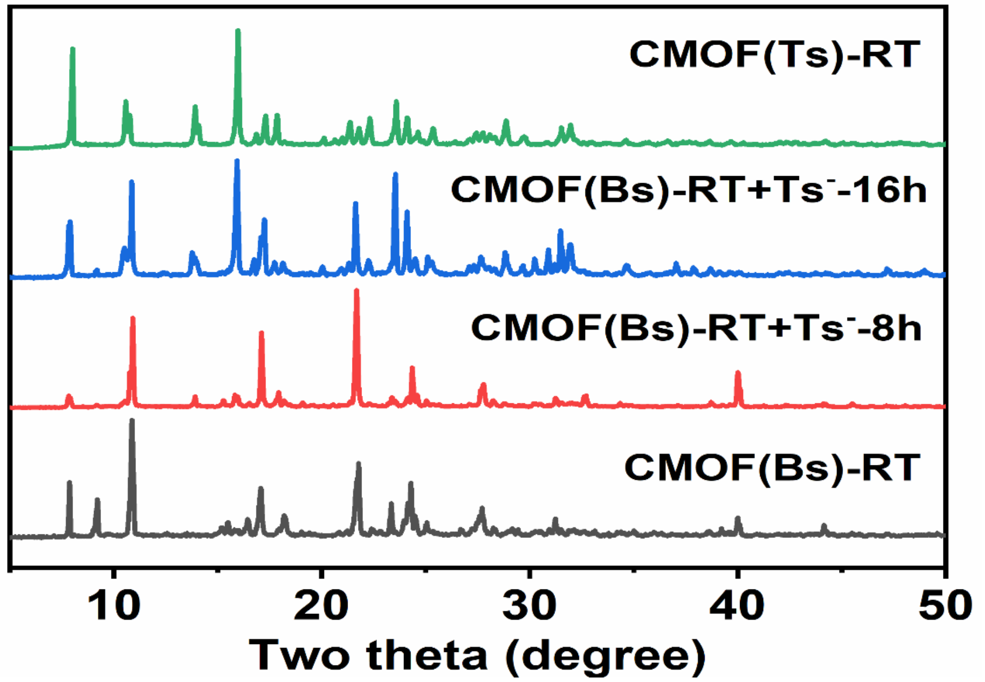

Figure S25. Time-dependent PXRD patterns of samples obtained from the anion-exchange experiments that CMOF(Bs)-RT was immersed in a 5-fold molar excess of aqueous Ts^{-}.

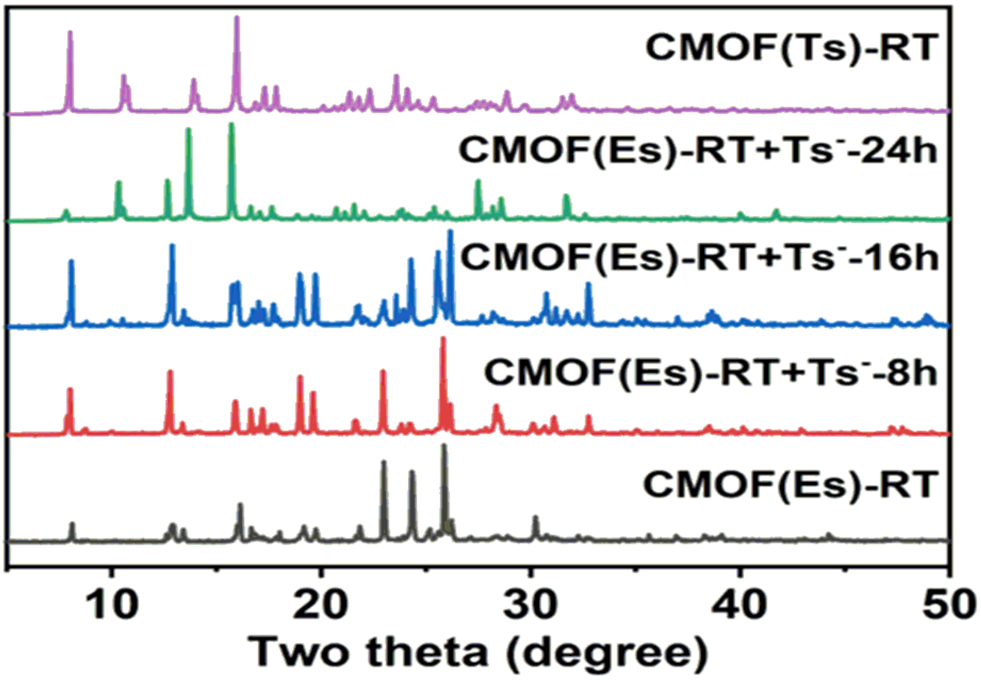

Figure S26. Time-dependent PXRD patterns of samples obtained from the anion-exchange experiments that CMOF(Es)-RT was immersed in a 5-fold molar excess of aqueous Ts^{-}.

8. Competition experiments in two-component mixtures at room temperature

Atrz (0.25 mmol) and $Cu(NO_3)_2$ (0.125 mmol) were added into three 10 mL aqueous solution containing Ts^- (0.25 mmol)/Bs^-(0.25 mmol), Ts^-(0.25 mmol)/Es^-(0.25 mmol), Ts^-(0.25 mmol)/Ps^-(0.25 mmol), respectively. The resulting solutions were placed under static conditions at room temperature for 24 h, affording blue crystals at the bottom of the solutions. After decanting the solution, the resultant crystals were washed thoroughly with deionized water, dried in vacuum to give the corresponding competition products.

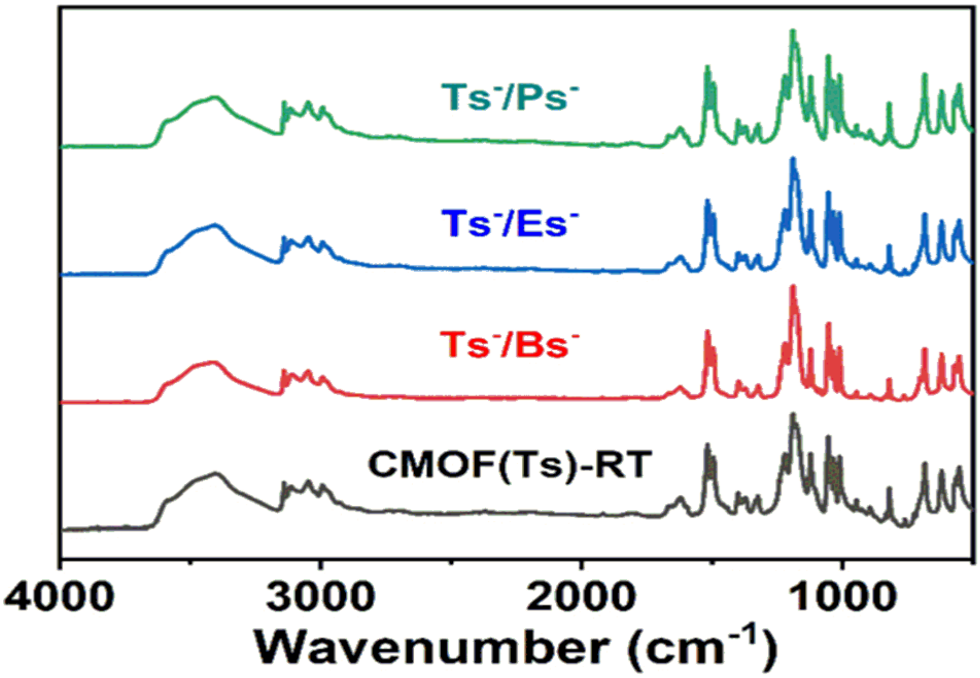

Figure S27. FT-IR spectra of CMOF(Ts)-RT and the samples obtained fromselective experiments of Ts^-/Bs^-, Ts^-/Es^- and Ts^-/Ps^- under the standard condition at room temperature.

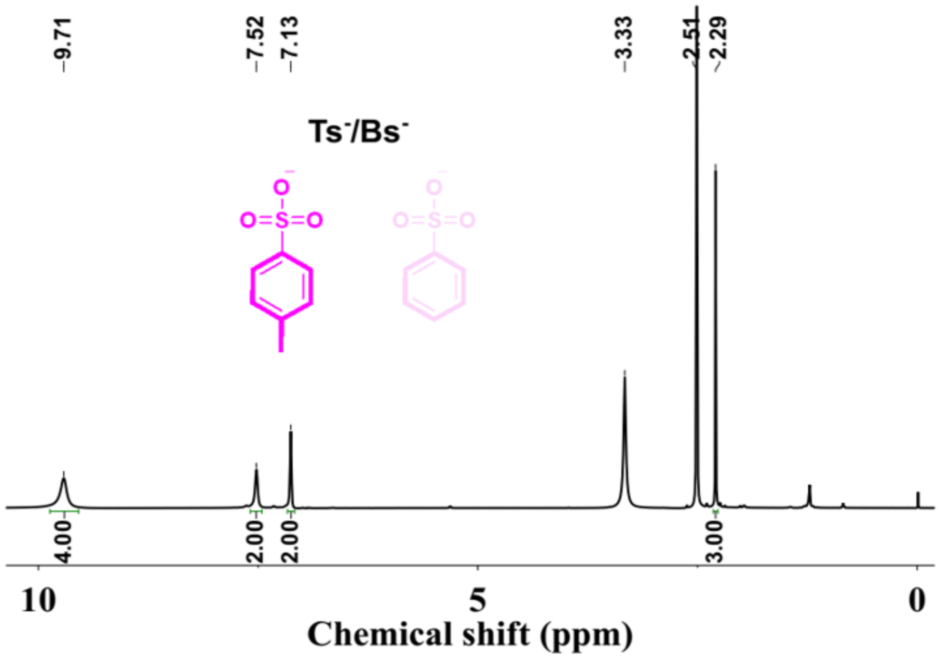

Figure S28. ^{1}H NMR spectrum of the sample (dissolved in DMSO-d_6) obtained from selective experiment between Ts^- and Bs^- in 1:1 ratio at room temperature. The selectivity of Ts^- over Bs^- was 100% calculated by the stoichiometric purity of Ts^- shown in the spectrum.

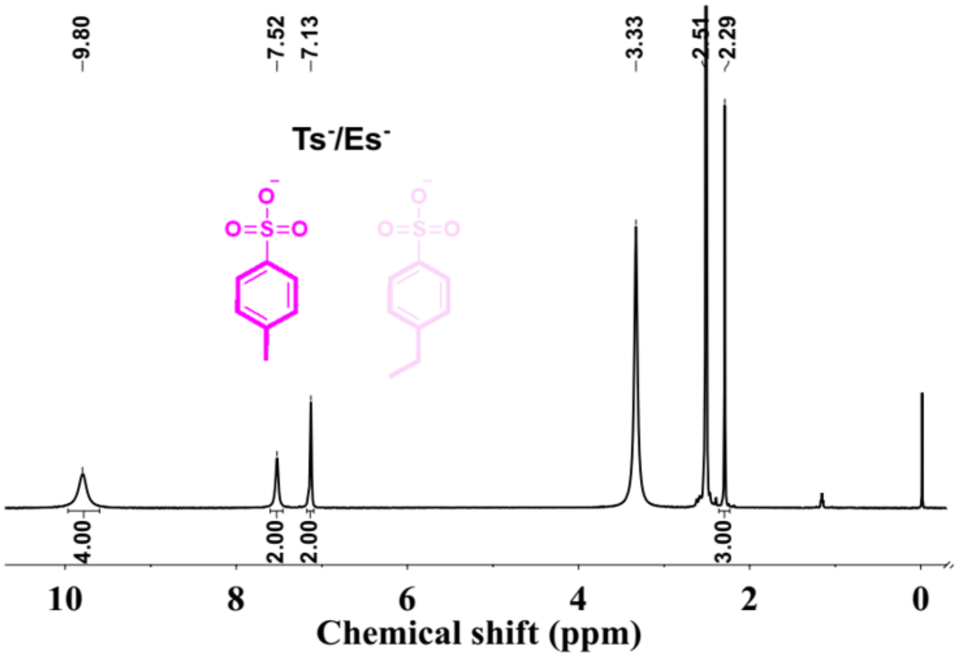

Figure S29. ^{1}H NMR spectrum of the sample (dissolved in DMSO-d_6) obtained from selective experiment between Ts^- and Es^- in 1:1 ratio at room temperature. The selectivity of Ts^- over Es^- was 100% calculated by the stoichiometric purity of Ts^- shown in the spectrum.

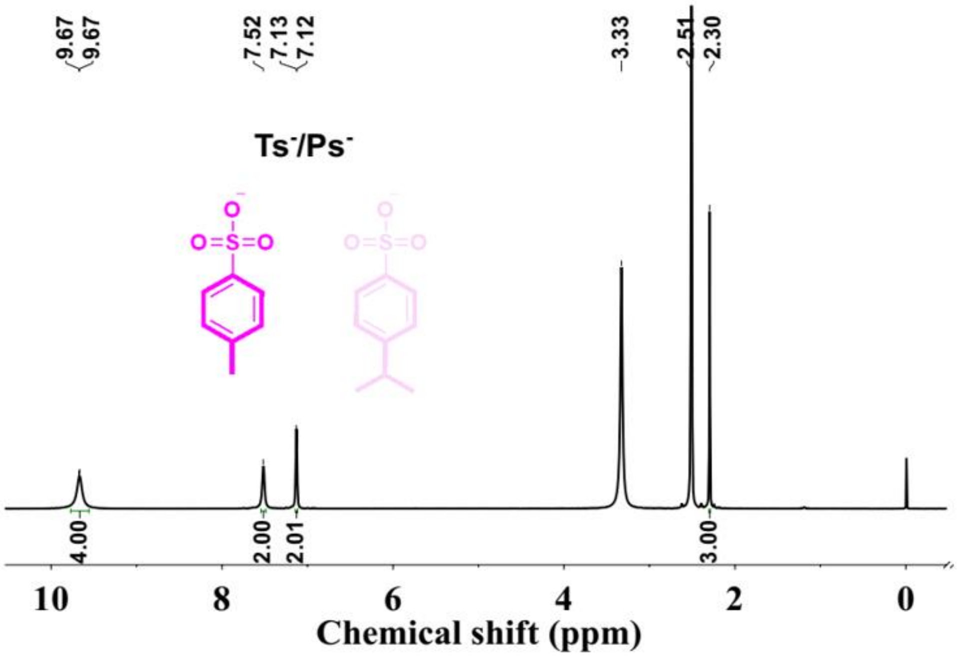

Figure S30.^{1}H NMR spectrum of the sample (dissolved in DMSO-d_6) obtained from selective experiment between Ts^- and Ps^- in 1:1 ratio at room temperature. The selectivity of Ts^- over Ps^- was 100% calculated by the stoichiometric purity of Ts^- shown in the spectrum.

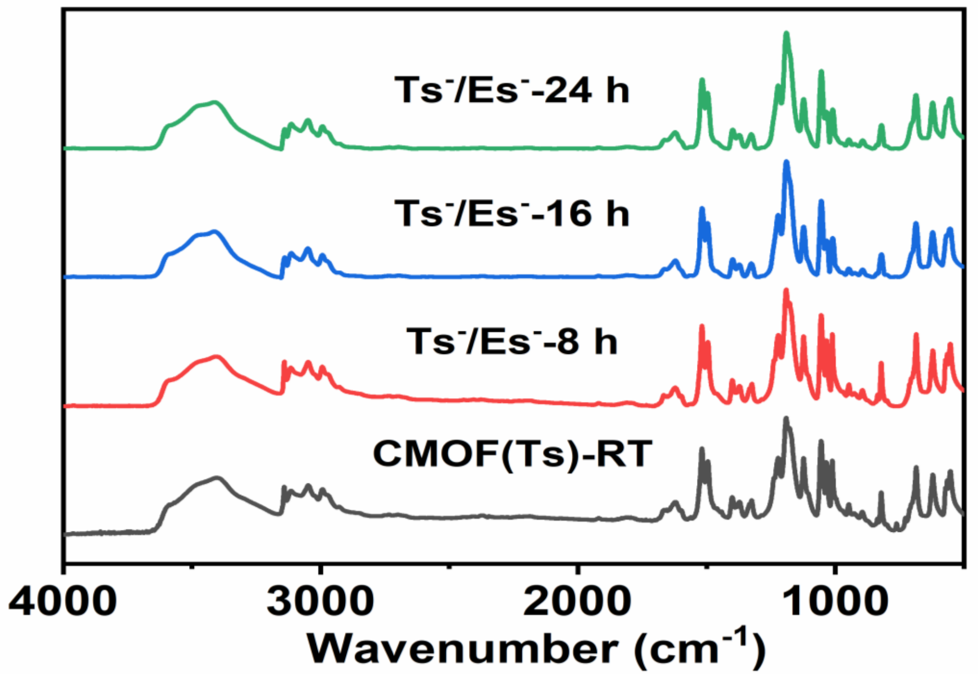

Figure S31. FT-IR spectra of CMOF(Ts)-RT and the samples obtained fromselective experiments of Ts^-/Es^- at different reaction times

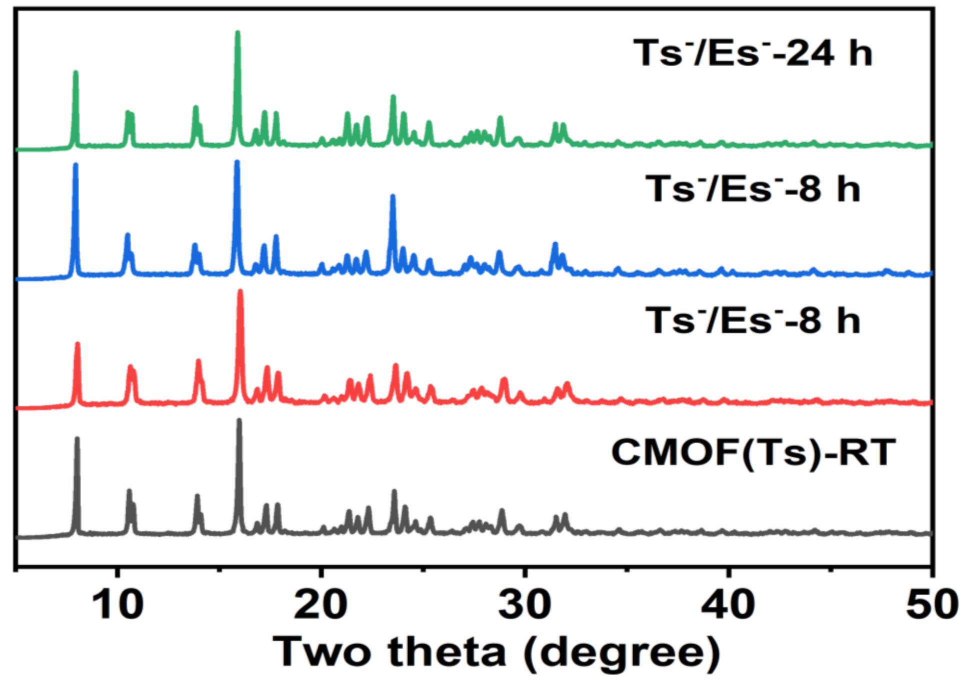

Figure S32. PXRD patterns of CMOF(Ts)-RT and the samples obtained fromselective experiments of Ts^-/Es^- at different reaction times

9. Preparation and structure characterizations of CMOFs obtained at 80°C

Preparation of CMOF(Ts)-80: 0.125 mmol $Cu(NO_3)_2$ were added into a 10 mL aqueous solution containing 0.25 mmol atrz and 0.25 mmol Na(Ts). The resulting clear solution was placed under static condition at 80 ^oC for 24 h, and then orange crystalline solids were produced at the bottom of the solution. After decanting the solution, the resultant crystals were washed thoroughly with deionized water, and dried in vacuum. The yield was 70.2% based on atrz. IR (KBr pellets, λ, cm^{-1}): 3080, 1615, 1510, 1378, 1325, 1188, 1121, 1060, 1008, 818, 680, 623, 557. ^1H NMR (600 MHz, DMSO-d_6): δ = 9.49 (s, 4H), 7.49 (d, J = 6.0 Hz, 2H), 7.12 (d, J = 6.0 Hz, 2H), 2.30 (s, 3H).

Preparation of CMOF(Bs)-80: 0.125 mmol $Cu(NO_3)_2$ were added into a 10 mL aqueous solution containing 0.25 mmol atrz and 0.25 mmol Na(Bs). The resulting clear solution was placed under static condition at 80 ^oC for 24 h, and then orange crystalline solids were produced at the bottom of the solution. After decanting the solution, the resultant crystals were washed thoroughly with deionized water, and dried in vacuum. The yield was 73.5% based on atrz. IR (KBr pellets, λ, cm^{-1}): 3094, 1624, 1496, 1444, 1378, 1325, 1220, 1177, 1117, 1047, 877, 752, 729, 697, 619, 558. ^1H NMR (600 MHz, DMSO-d_6): δ = 9.48 (s, 3H), 7.61 (d, *J* = 6.0 Hz, 2H), 7.33 – 7.31 (m, 3H).

Preparation of CMOF(Es)-80: 0.125 mmol $Cu(NO_3)_2$ were added into a 10 mL aqueous solution containing 0.25 mmol atrz and 0.25 mmol Na(Es). The resulting clear solution was placed under static condition at 80 ^oC for 24 h, and then orange crystalline solids were produced at the bottom of the solution. After decanting the solution, the resultant crystals were washed thoroughly with deionized water, and dried in vacuum. The yield was 76.8% based on atrz. IR (KBr pellets, λ, cm^{-1}): 3091, 1629, 1490, 1380, 1323, 1179, 1051, 1008, 832, 681, 619, 553. ^1H NMR (600 MHz, DMSO-d_6): δ = 9.49 (s, 4H), 7.52 (d, J = 6.0 Hz, 2H), 7.15 (d, J = 6.0 Hz, 2H), 2.60 – 2.58 (m, 2H), 1.18 – 1.16 (m, 3H).

Preparation of CMOF(Ps)-80: 0.125 mmol $Cu(NO_3)_2$ were added into a 10 mL aqueous solution containing 0.25 mmol atrz and 0.25 mmol sodium 4-propan-2-ylbenzenesulfonate [Na(Ps)]. The resulting clear solution was placed under static condition at 80 °C for 24 h, and then orange crystalline solids were produced at the bottom of the solution. After decanting the solution, the resultant crystals were washed thoroughly with deionized water, and dried in vacuum. The yield was 78.6% based on atrz. IR (KBr pellets, λ, cm^{-1}): 3089, 1636, 1497, 1382, 1325, 1179, 1051, 1006, 834, 674, 622, 583, 551. ^{1}H NMR (600 MHz, DMSO-d_6): δ = 9.49 (s, 4H), 7.55 - 7.51 (m, 2H), 7.19 (d, J = 6.0 Hz, 2H), 2.88 (s, 1H), 1.20 (s, 6H).

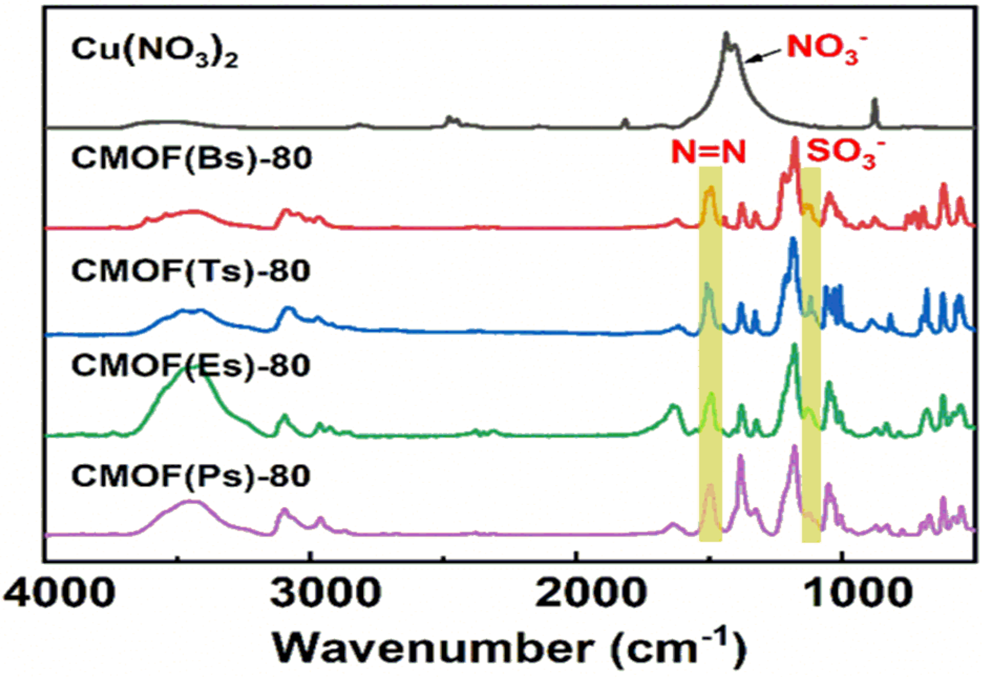

Figure S33. FT-IR spectra of CMOF(Bs)-80, CMOF(Ts)-80, CMOF(Es)-80 and CMOF(Ps)-80.

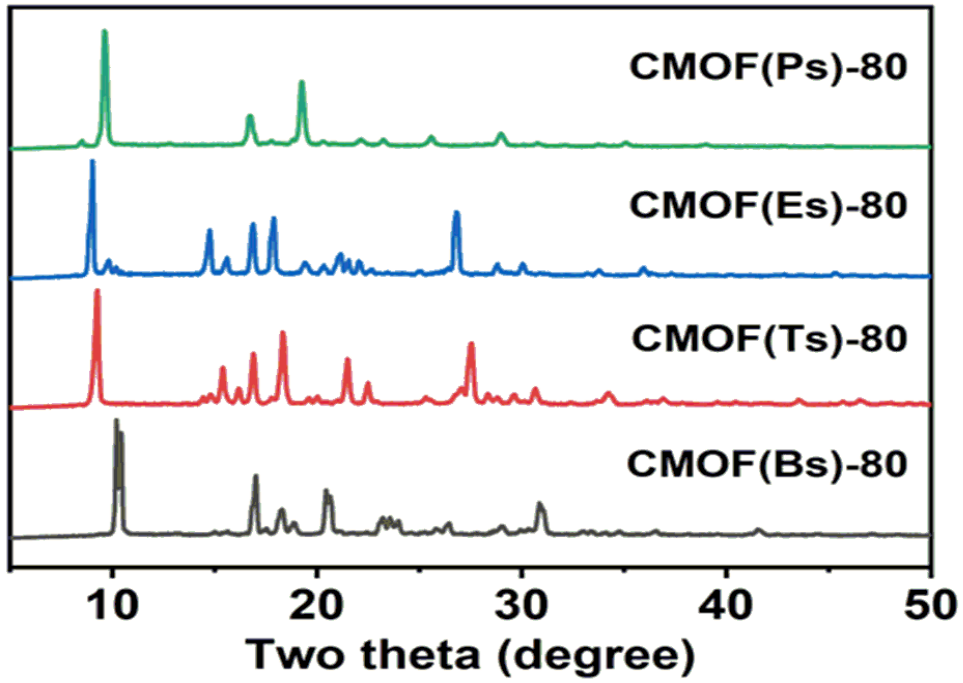

Figure S34. PXRD patterns of CMOF(Bs)-80, CMOF(Ts)-80, CMOF(Es)-80 and CMOF(Ps)-80.

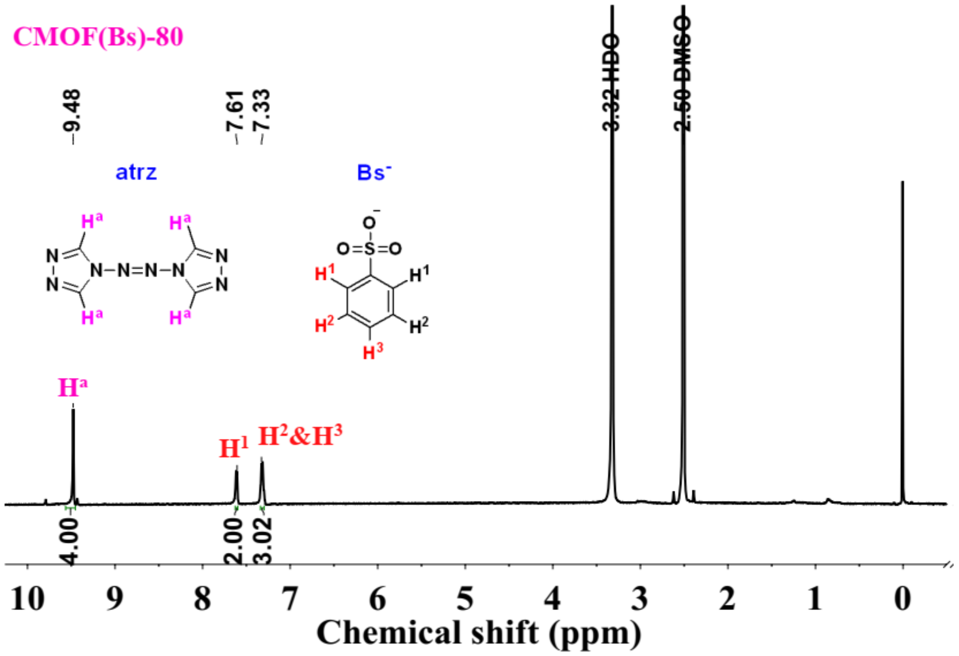

Figure S35. ^{1}H-NMR spectrum of CMOF(Bs)-80 dissolved in DMSO-d_6.

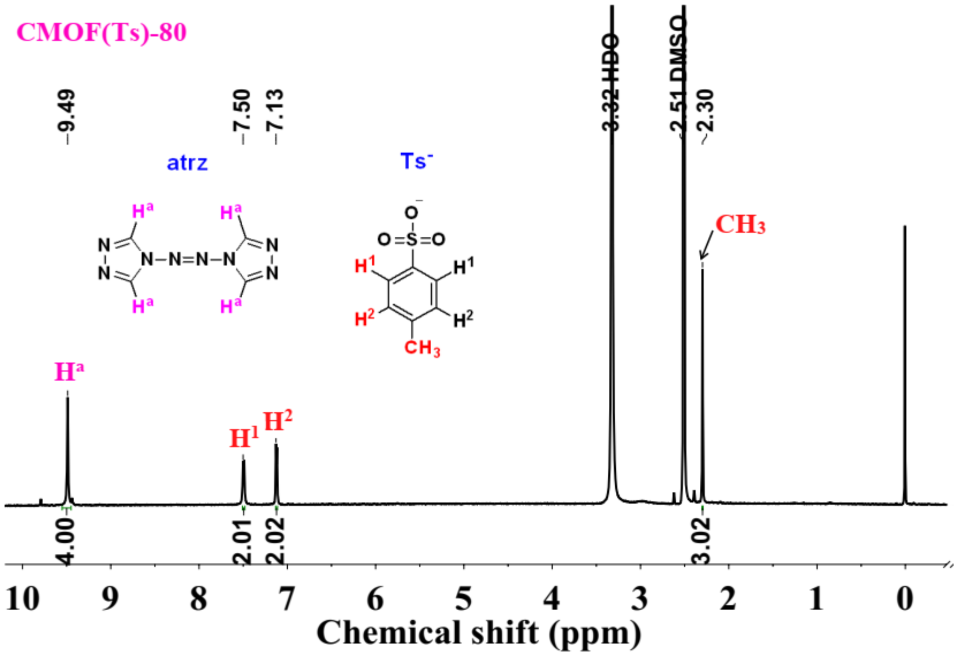

Figure S36.^{1}H-NMR spectrum of CMOF(Ts)-80 dissolved in DMSO-d_6.

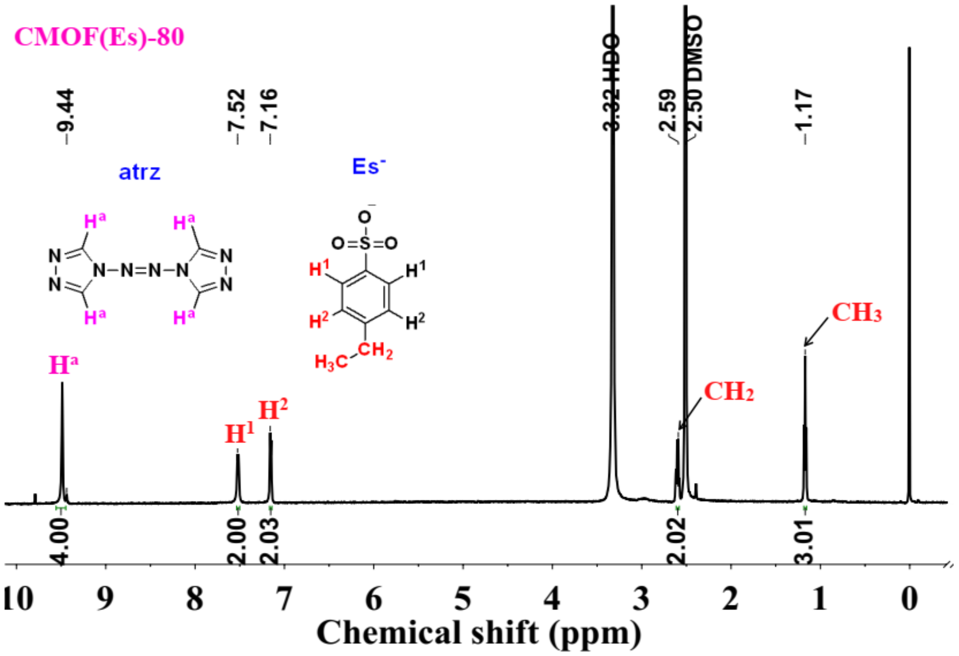

Figure S37. ^{1}H-NMR spectrum of CMOF(Es)-80dissolved in DMSO-d_6.

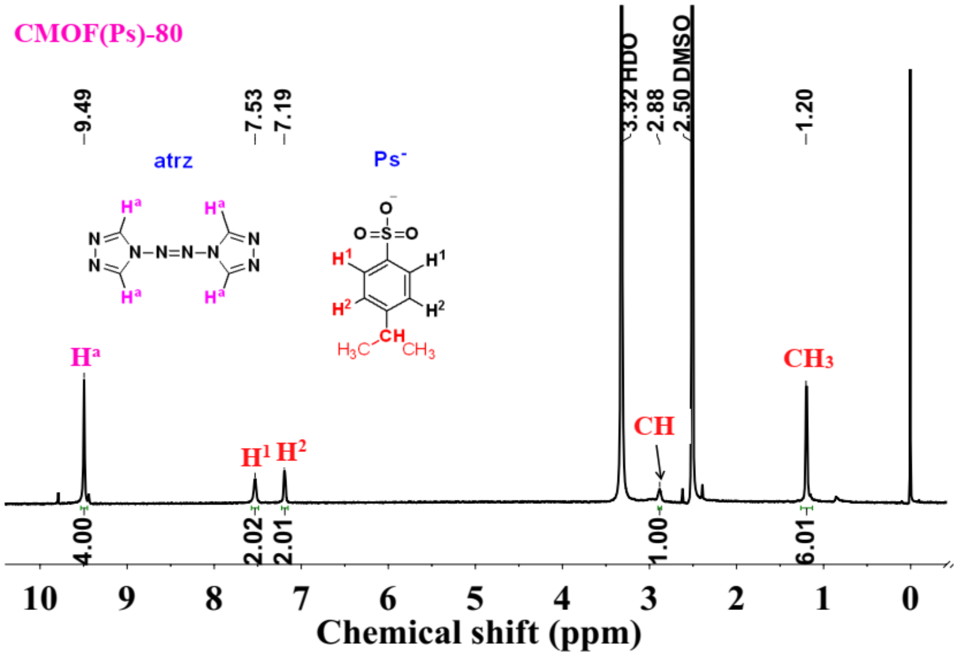

Figure S38. ^{1}H-NMR spectrum of CMOF(Ps)-80 dissolved in DMSO-d_6.

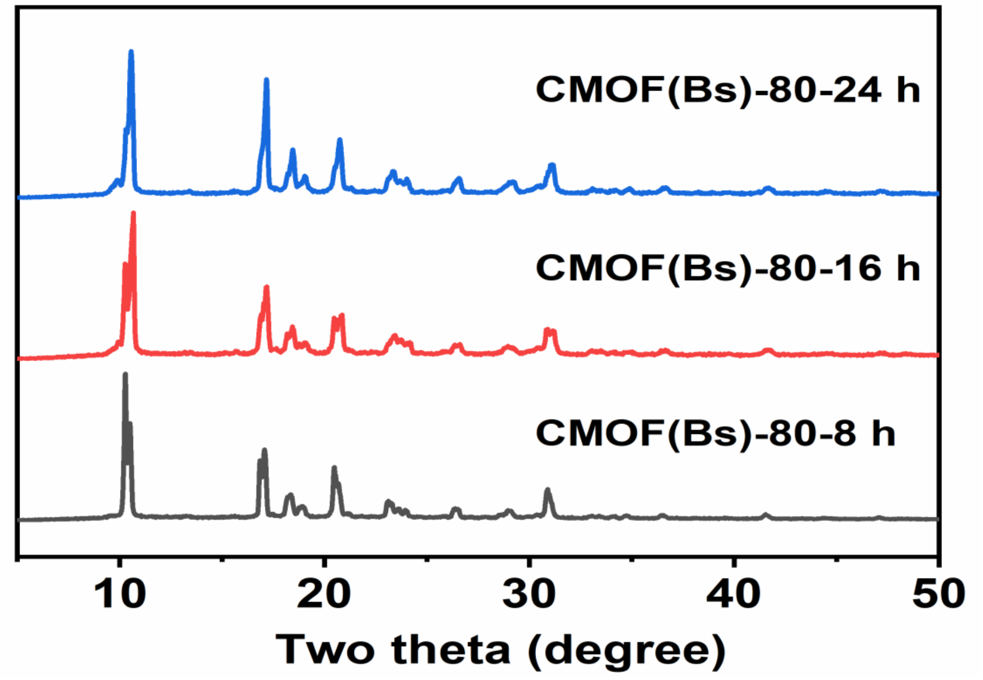

Figure S39. PXRD patterns of the samples obtained from the reaction of 0.25 mmol Bs^-, 0.25 mmol atrz and 0.125 mmol $Cu(NO_3)_2$at different reaction times at 80 °C.

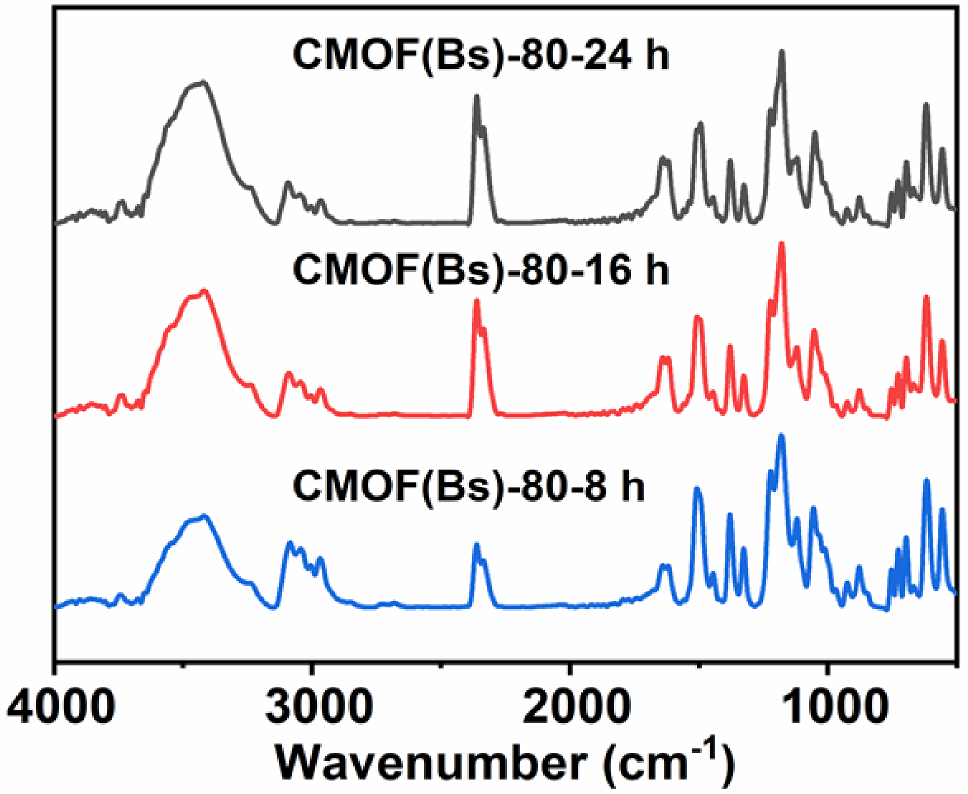

Figure S40. IR spectra of CMOF(Bs)-80 and the samples obtained from the reaction of 0.25 mmol Bs^-, 0.25 mmol atrz and 0.125 mmol $Cu(NO_3)_2$at different reaction times at 80 °C.

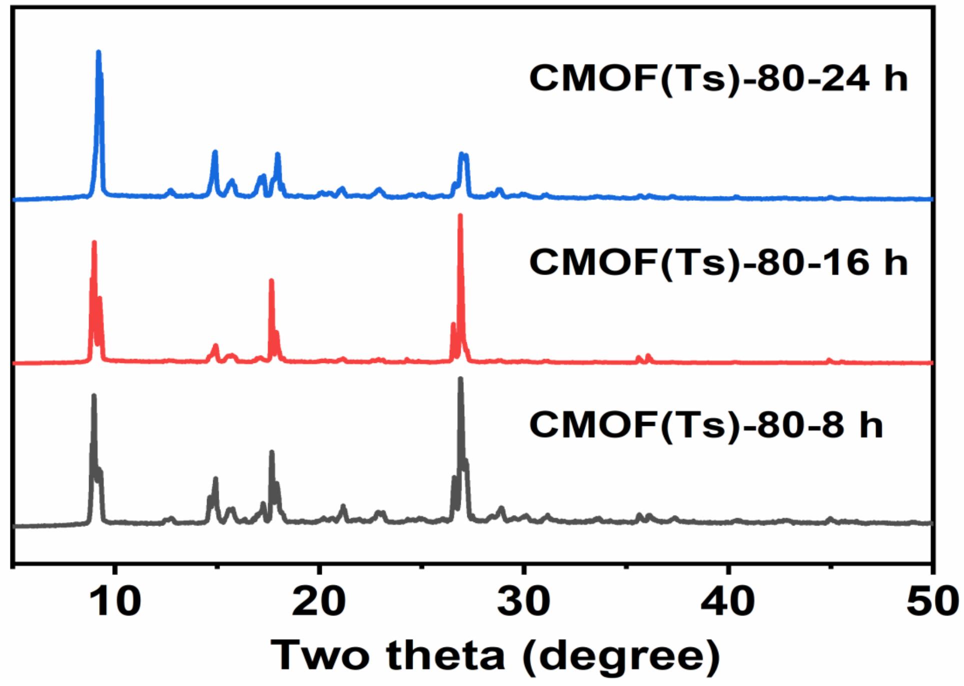

Figure S41. PXRD patterns of the samples obtained from the reaction of 0.25 mmol Ts^-, 0.25 mmol atrz and 0.125 mmol $Cu(NO_3)_2$at different reaction times at 80 °C.

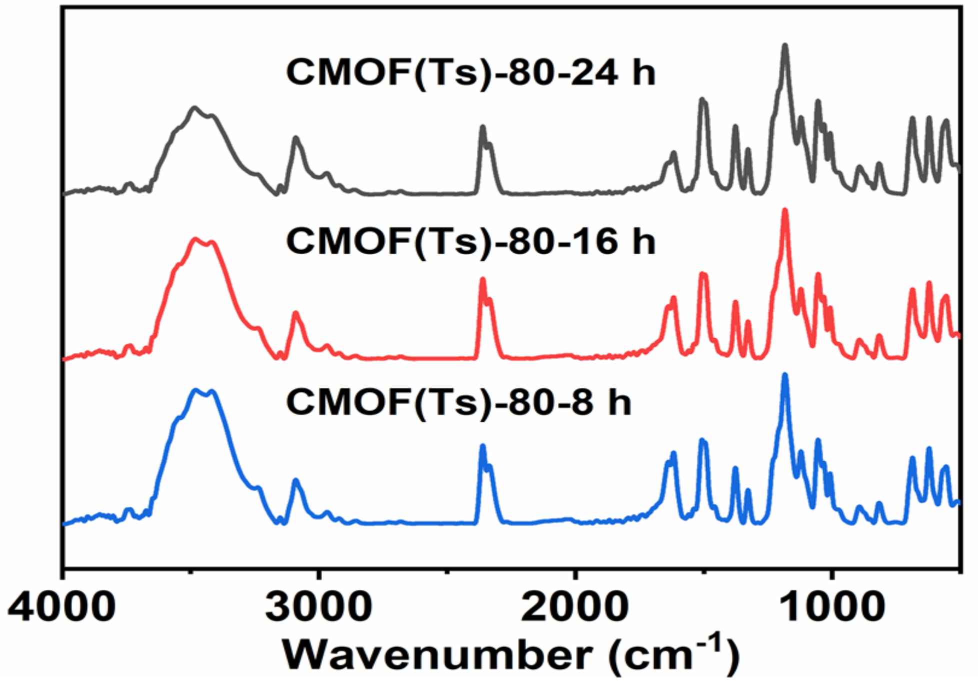

Figure S42. IR spectra of CMOF(Ts)-80 and the samples obtained from the reaction of 0.25 mmol Ts^-, 0.25 mmol atrz and 0.125 mmol $Cu(NO_3)_2$at different reaction times at 80 °C.

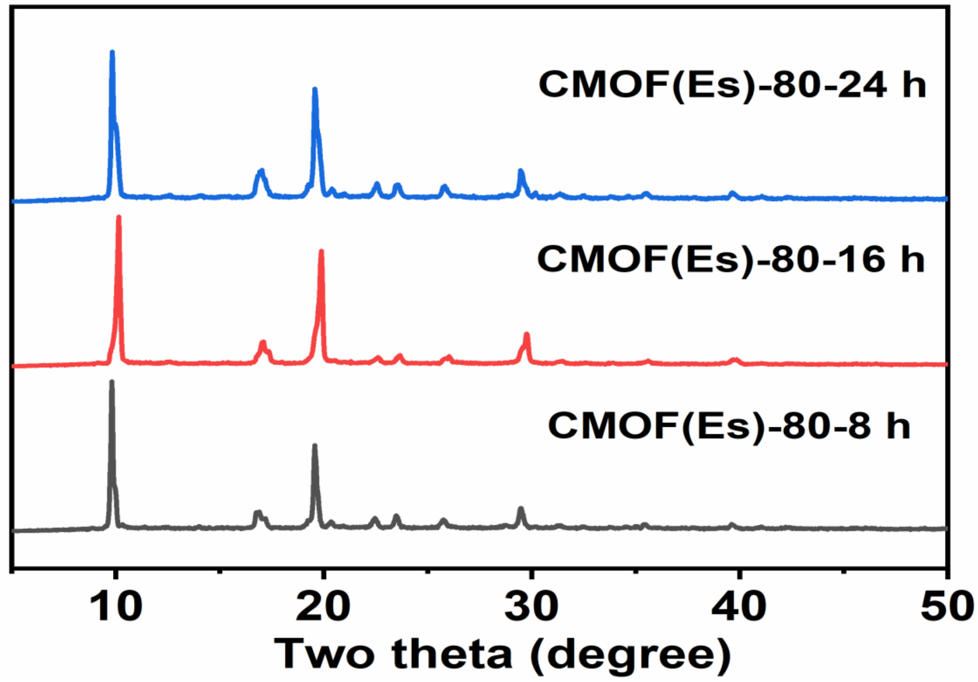

Figure S43. PXRD patterns of CMOF(Es)-80 and the samples obtained from the reaction of 0.25 mmol Es^-, 0.25 mmol atrz and 0.125 mmol $Cu(NO_3)_2$at different reaction times at 80 °C.

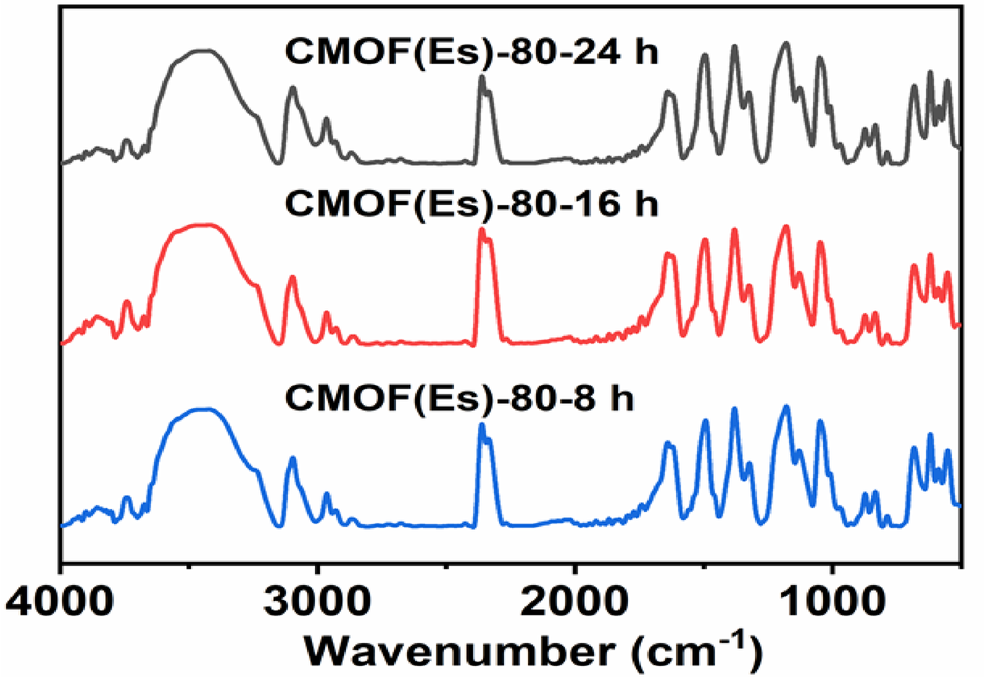

Figure S44. IR spectra of CMOF(Es)-80 and the samples obtained from the reaction of 0.25 mmol Es$^-$, 0.25 mmol atrz and 0.125 mmol $Cu(NO_3)_2$at different reaction times at 80 ^oC.

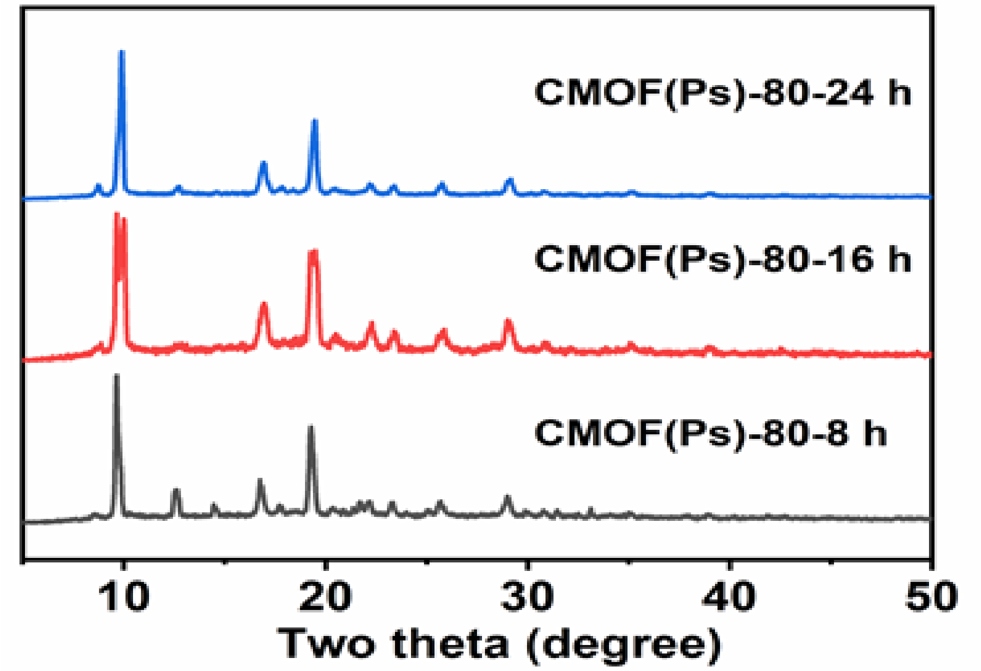

Figure S45. PXRD patterns of CMOF(Ps)-80 and the samples obtained from the reaction of 0.25 mmol Ps$^-$, 0.25 mmol atrz and 0.125 mmol $Cu(NO_3)_2$at different reaction times at 80 ^oC.

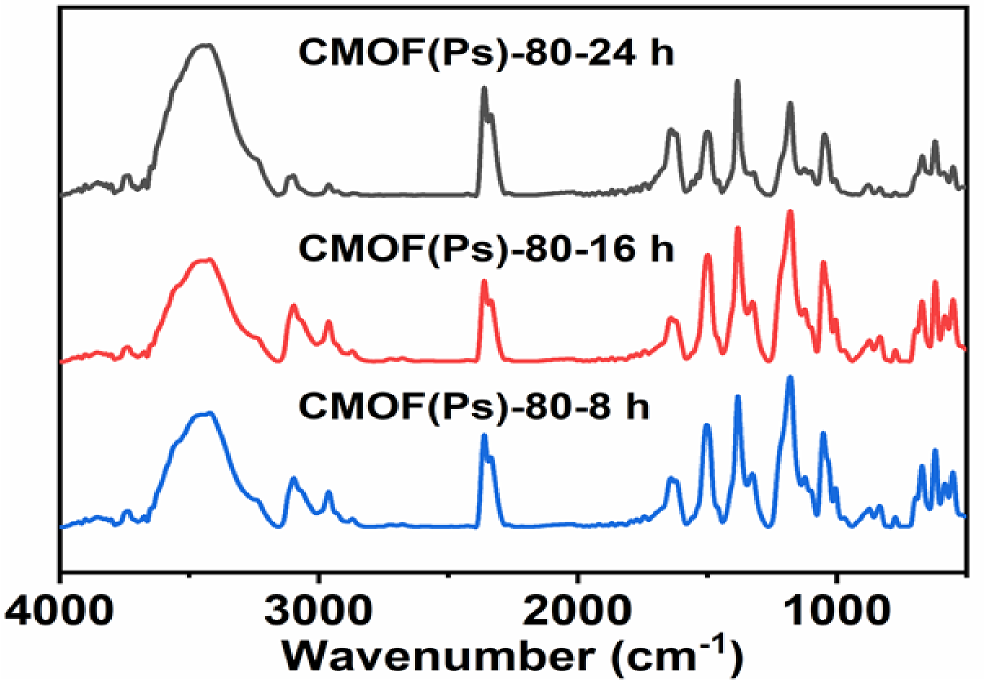

Figure S46. IR spectra of CMOF(Ps)-80 and the samples obtained from the reaction of 0.25 mmol Ps^-, 0.25 mmol atrz and 0.125 mmol $Cu(NO_3)_2$at different reaction times at 80 °C.

10. Competition experiments in two-component mixtures at 80 °C

Atrz (0.25 mmol) and $Cu(NO_3)_2$ (0.125 mmol) were added into three 10 mL aqueous solution containing Ts^- (0.25 mmol)/Bs^- (0.25 mmol), Ts^- (0.25 mmol)/Es^- (0.25 mmol), Ts^- (0.25 mmol)/Ps^- (0.25 mmol), respectively. The resulting solutions were placed under static conditions at 80 °C for 24 h, affording orange crystals at the bottom of the solutions. After decanting the solution, the resultant crystals were washed thoroughly with deionized water, dried in vacuum to give the corresponding competition products.

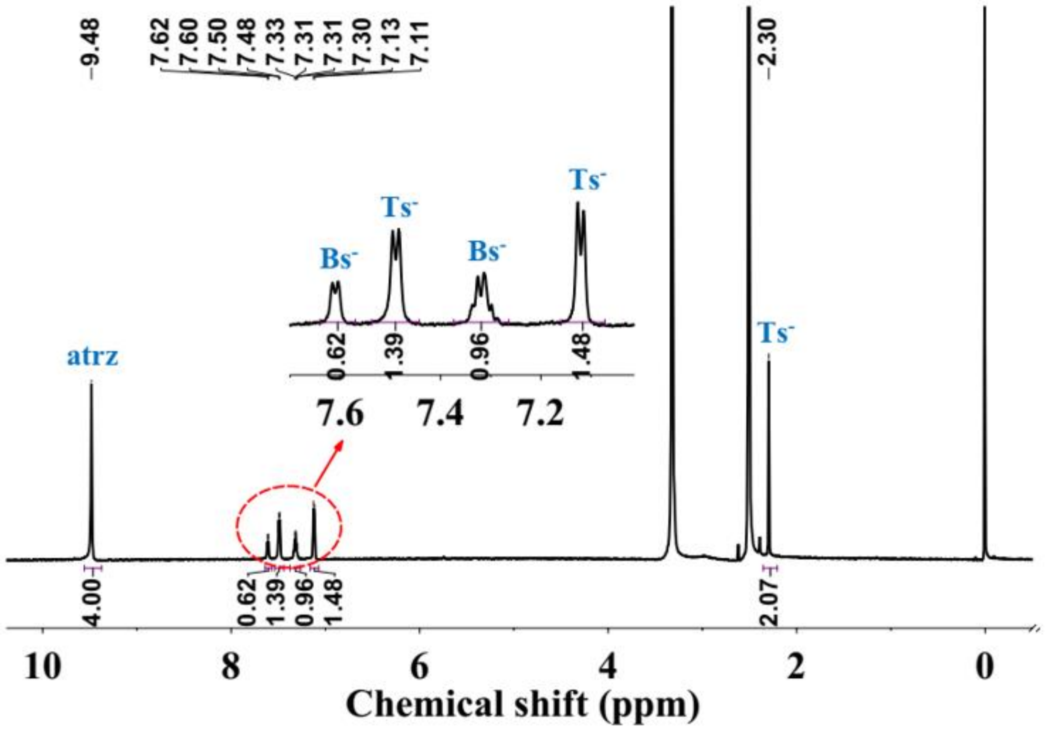

Figure S47. ^{1}H-NMR spectrum of the sample (dissolved in DMSO-d_6) obtained from selective experiments between Ts^- and Bs^- in 1:1 ratio at 80 ^oC. The selectivity of Ts^- over Bs^- was 69% [1.39/(0.62+1.39)] by intergrating the peaks of Ts^- vs Bs^- (pointed in the red cycle) in the spectrum.

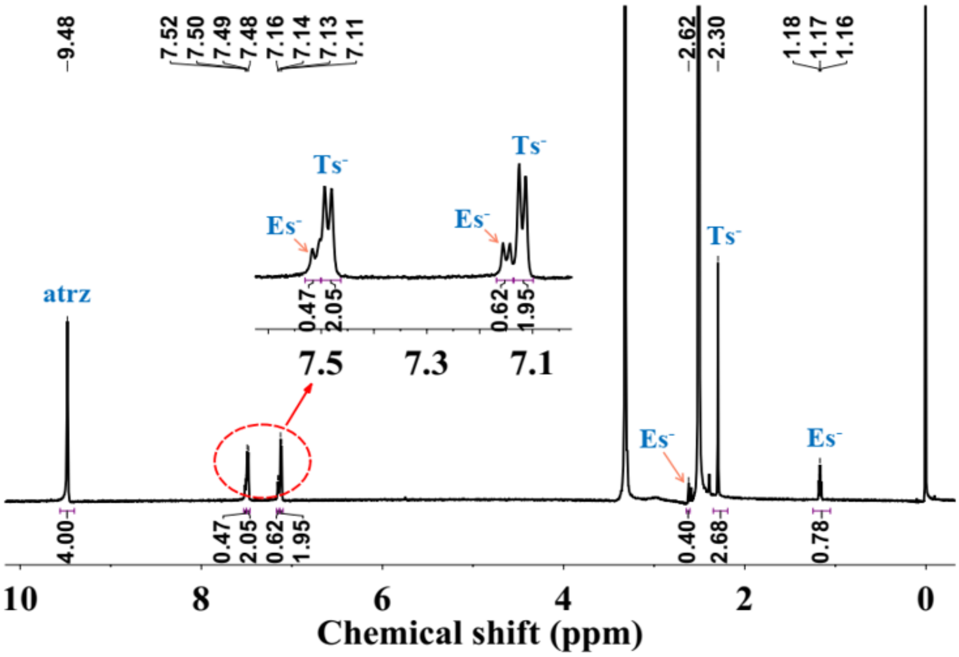

Figure S48. ^{1}H-NMR spectrum of the sample (dissolved in DMSO-d_6) obtained from selective experiments between Ts^- and Es^- in 1:1 ratio at 80 °C. The selectivity of Ts^- over Es^- was 80% [2.05/(2.05+0.470)] by intergrating the peaks of Ts^- vs Es^- (pointed in the red cycle) in the spectrum.

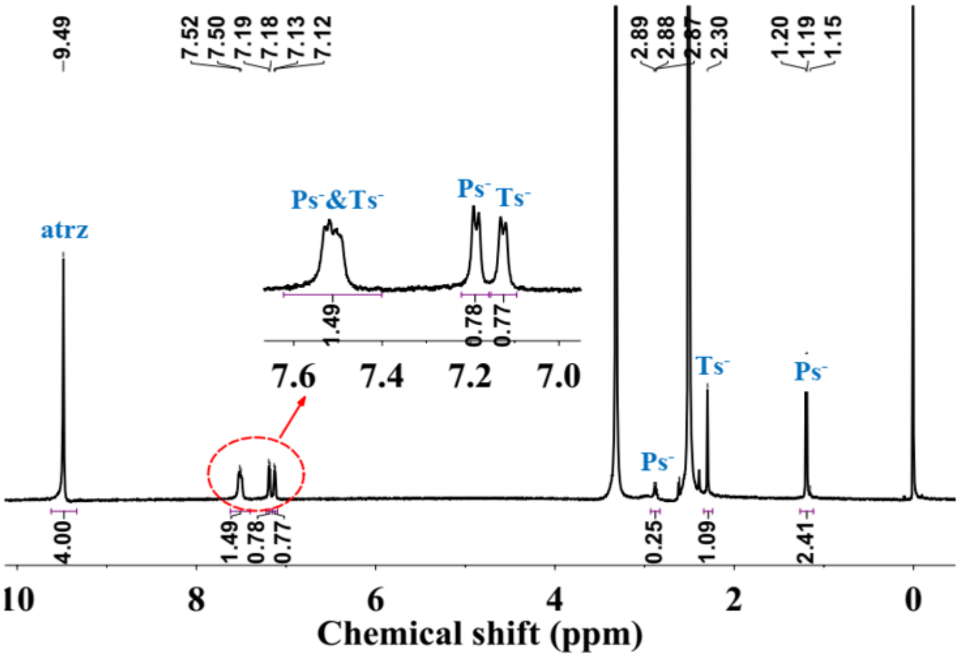

Figure S49. ^{1}H-NMR spectrum of the sample (dissolved in DMSO-d_6) obtained from selective experiments between Ts^- and Ps^- in 1:1 ratio at 80 ^{o}C. The selectivity of Ts^- over Ps^- was 50% [0.77/(0.77+0.78)] by intergrating the peaks of Ts^- vs Ps^- (pointed in the red cycle) in the spectrum.

11. Competition experiments in multicomponent mixtures at room temperature.

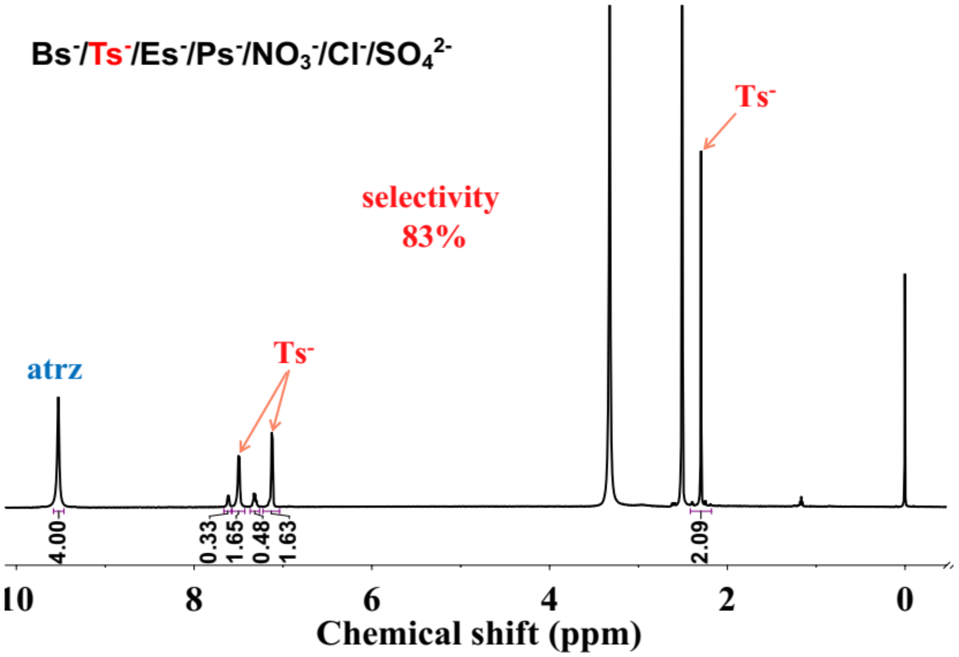

Figure S50.^{1}H NMR (solvent: d_6-DMSO) spectra of CMOF(Ts)-RT, and the samples obtained fromthe multicomponent selective experiment ($Bs^-/Ts^-/Es^-/Ps^-/NO_3^-/Cl^-/SO_4^{2}$) at room temperature. The selectivity of Ts^- over Bs^- was 83% calculated by the stoichiometric purity of Ts^- shown in the spectrum.

12. Recovery and Reusability.

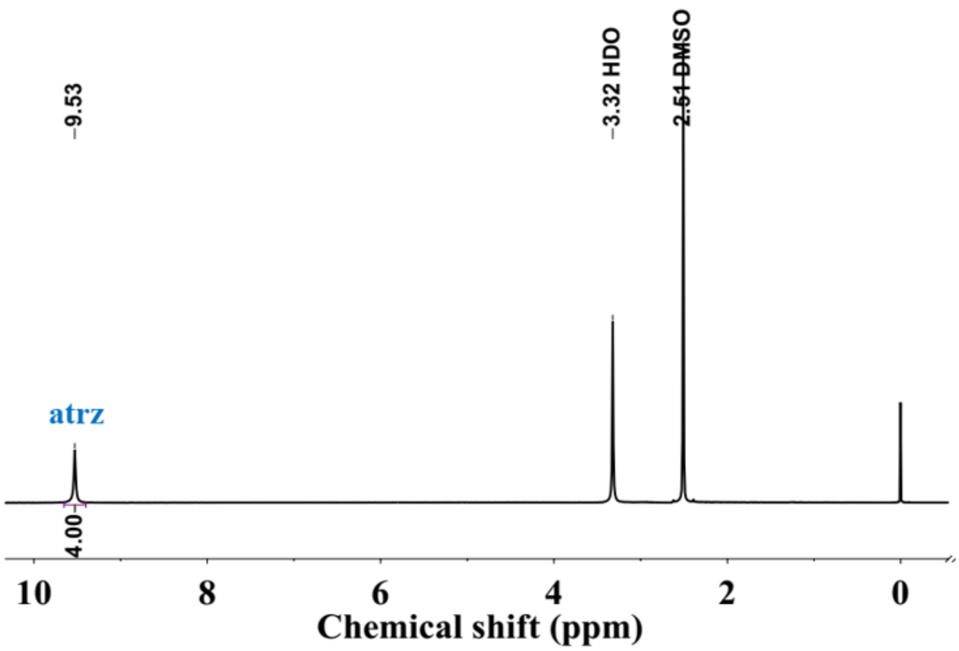

Figure S51.^{1}H-NMR spectrum of the sample (dissolved in DMSO-d_6) obtained from the anion-exchange experiment between CMOF(Ts)-RT and NO_3^-after 72 h.

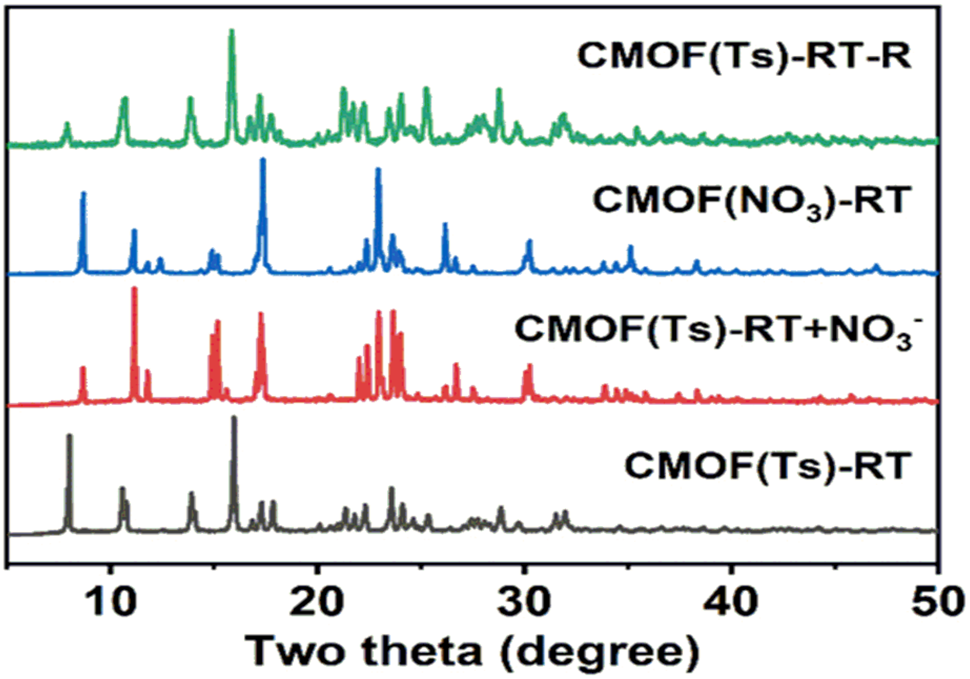

Figure S52. PXRD patterns of samples obtainedfrom the anion-exchange-recycle experiments of CMOF(Ts)-RT.

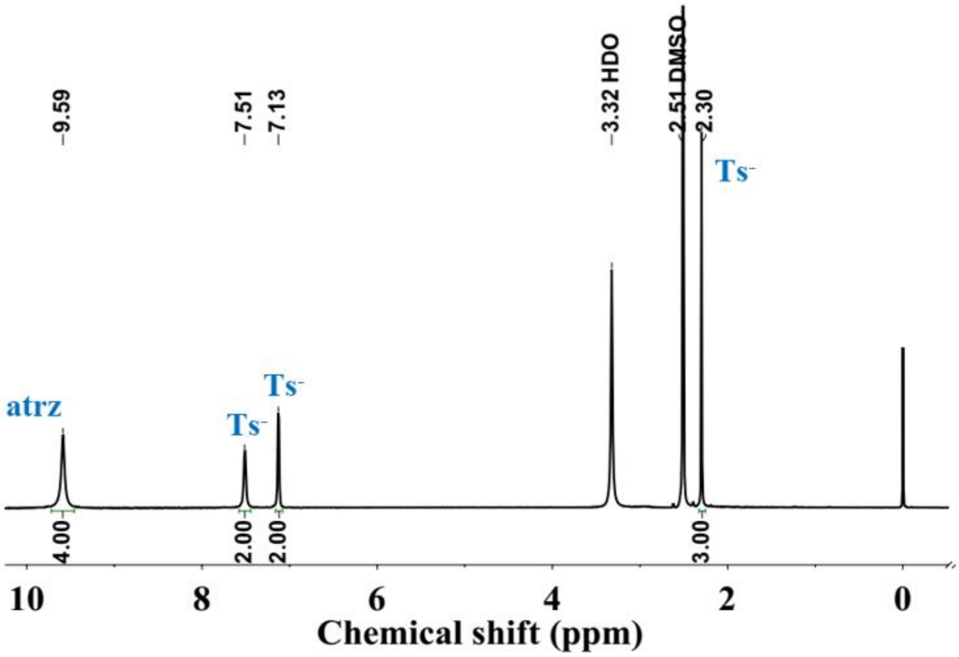

Figure S53. ^{1}H-NMR spectrum of CMOF(Ts)-RT-R dissolved in DMSO-$d_6$recovered from the anion-exchange-recycle experiments.

13. Electrostatic potential (ESP) of sulfonate anions

```
================= Summary of surface analysis =================

Volume:  1142.70394 Bohr^3  ( 169.33129 Angstrom^3)
Estimated density according to mass and volume (M/V):    1.5413 g/cm^3
Minimal value:   -137.94583 kcal/mol   Maximal value:    -39.68271 kcal/mol
Overall surface area:         603.87491 Bohr^2  ( 169.10222 Angstrom^2)
Positive surface area:          0.00000 Bohr^2  (   0.00000 Angstrom^2)
Negative surface area:        603.87491 Bohr^2  ( 169.10222 Angstrom^2)
Overall average value:   -0.14342833 a.u. (    -90.00271 kcal/mol)
Positive average value:           NaN a.u. (          NaN kcal/mol)
Negative average value:  -0.14342833 a.u. (    -90.00271 kcal/mol)
Overall variance (sigma^2_tot):  0.00234726 a.u.^2 (   924.27842 (kcal/mol)^2)
Positive variance:        0.00000000 a.u.^2 (      0.00000 (kcal/mol)^2)
Negative variance:        0.00234726 a.u.^2 (    924.27842 (kcal/mol)^2)
Balance of charges (nu):   0.00000000
Product of sigma^2_tot and nu:   0.00000000 a.u.^2 (    0.00000 (kcal/mol)^2)
Internal charge separation (Pi):   0.04413638 a.u. (     27.69602 kcal/mol)
Molecular polarity index:   3.90288333 eV (     90.00271 kcal/mol)
Nonpolar surface area (|ESP| <= 10 kcal/mol):      0.00 Angstrom^2  (  0.00 %)
Polar surface area (|ESP| > 10 kcal/mol):       169.10 Angstrom^2  (100.00 %)
```

Figure S54. Summary of surface analysis of Bs- anion.

```
================= Summary of surface analysis =================

Volume:  1275.84346 Bohr^3  ( 189.06054 Angstrom^3)
Estimated density according to mass and volume (M/V):    1.5036 g/cm^3
Minimal value:   -140.37685 kcal/mol   Maximal value:    -36.43045 kcal/mol
Overall surface area:         668.64984 Bohr^2  ( 187.24105 Angstrom^2)
Positive surface area:          0.00000 Bohr^2  (   0.00000 Angstrom^2)
Negative surface area:        668.64984 Bohr^2  ( 187.24105 Angstrom^2)
Overall average value:   -0.13529792 a.u.  (    -84.90079 kcal/mol)
Positive average value:          NaN a.u.  (          NaN kcal/mol)
Negative average value:  -0.13529792 a.u.  (    -84.90079 kcal/mol)
Overall variance (sigma^2_tot):  0.00289998 a.u.^2 (  1141.92084 (kcal/mol)^2)
Positive variance:        0.00000000 a.u.^2 (      0.00000 (kcal/mol)^2)
Negative variance:        0.00289998 a.u.^2 (   1141.92084 (kcal/mol)^2)
Balance of charges (nu):   0.00000000
Product of sigma^2_tot and nu:   0.00000000 a.u.^2 (    0.00000 (kcal/mol)^2)
Internal charge separation (Pi):   0.04862806 a.u.  (     30.51460 kcal/mol)
Molecular polarity index:   3.68164350 eV (     84.90079 kcal/mol)
Nonpolar surface area (|ESP| <= 10 kcal/mol):      0.00 Angstrom^2  (  0.00 %)
Polar surface area (|ESP| > 10 kcal/mol):       187.24 Angstrom^2  (100.00 %)
```

Figure S55. Summary of surface analysis of Ts- anion.

```
================= Summary of surface analysis =================

Volume:  1421.12333 Bohr^3  ( 210.58880 Angstrom^3)
Estimated density according to mass and volume (M/V):    1.4605 g/cm^3
Minimal value:   -136.78536 kcal/mol   Maximal value:    -29.50260 kcal/mol
Overall surface area:         732.83007 Bohr^2  ( 205.21335 Angstrom^2)
Positive surface area:          0.00000 Bohr^2  (   0.00000 Angstrom^2)
Negative surface area:        732.83007 Bohr^2  ( 205.21335 Angstrom^2)
Overall average value:   -0.12835466 a.u.  (    -80.54384 kcal/mol)
Positive average value:          NaN a.u.  (          NaN kcal/mol)
Negative average value:  -0.12835466 a.u.  (    -80.54384 kcal/mol)
Overall variance (sigma^2_tot):  0.00320886 a.u.^2 (  1263.54940 (kcal/mol)^2)
Positive variance:        0.00000000 a.u.^2 (      0.00000 (kcal/mol)^2)
Negative variance:        0.00320886 a.u.^2 (   1263.54940 (kcal/mol)^2)
Balance of charges (nu):   0.00000000
Product of sigma^2_tot and nu:   0.00000000 a.u.^2 (    0.00000 (kcal/mol)^2)
Internal charge separation (Pi):   0.05132213 a.u.  (     32.20515 kcal/mol)
Molecular polarity index:   3.49270804 eV (     80.54384 kcal/mol)
Nonpolar surface area (|ESP| <= 10 kcal/mol):      0.00 Angstrom^2  (  0.00 %)
Polar surface area (|ESP| > 10 kcal/mol):       205.21 Angstrom^2  (100.00 %)
```

Figure S56. Summary of surface analysis of Es- anion.
